# The complex genetics of gait speed: genome-wide meta-analysis approach

**DOI:** 10.18632/aging.101151

**Published:** 2017-01-10

**Authors:** Dan Ben-Avraham, David Karasik, Joe Verghese, Kathryn L. Lunetta, Jennifer A. Smith, John D. Eicher, Rotem Vered, Joris Deelen, Alice M. Arnold, Aron S. Buchman, Toshiko Tanaka, Jessica D. Faul, Maria Nethander, Myriam Fornage, Hieab H. Adams, Amy M. Matteini, Michele L. Callisaya, Albert V. Smith, Lei Yu, Philip L. De Jager, Denis A. Evans, Vilmundur Gudnason, Albert Hofman, Alison Pattie, Janie Corley, Lenore J. Launer, Davis S. Knopman, Neeta Parimi, Stephen T. Turner, Stefania Bandinelli, Marian Beekman, Danielle Gutman, Lital Sharvit, Simon P. Mooijaart, David C. Liewald, Jeanine J. Houwing-Duistermaat, Claes Ohlsson, Matthijs Moed, Vincent J. Verlinden, Dan Mellström, Jos N. van der Geest, Magnus Karlsson, Dena Hernandez, Rebekah McWhirter, Yongmei Liu, Russell Thomson, Gregory J. Tranah, Andre G. Uitterlinden, David R. Weir, Wei Zhao, John M. Starr, Andrew D. Johnson, M. Arfan Ikram, David A. Bennett, Steven R. Cummings, Ian J. Deary, Tamara B. Harris, Sharon L. R. Kardia, Thomas H. Mosley, Velandai K. Srikanth, Beverly G. Windham, Ann B. Newman, Jeremy D. Walston, Gail Davies, Daniel S. Evans, Eline P. Slagboom, Luigi Ferrucci, Douglas P. Kiel, Joanne M. Murabito, Gil Atzmon

**Affiliations:** ^1^ Department of Medicine and Genetics Albert Einstein College of Medicine, Bronx, NY 10461, USA; ^2^ Institute for Aging Research, Hebrew SeniorLife, Department of Medicine, Beth Israel Deaconess Medical Center and Harvard Medical School, Boston, MA 02131, USA; ^3^ Faculty of Medicine in the Galilee, Bar-Ilan University, Safed, Israel; ^4^ Integrated Divisions of Cognitive & Motor Aging (Neurology) and Geriatrics (Medicine), Montefiore-Einstein Center for the Aging Brain, Albert Einstein College of Medicine, Bronx, NY 10461, USA; ^5^ The National Heart Lung and Blood Institute's Framingham Heart Study, Framingham, MA 01702, USA; ^6^ Department of Biostatistics, Boston University School of Public Health, Boston, MA 02118, USA; ^7^ Department of Epidemiology, School of Public Health, University of Michigan, Ann Arbor, MI 48109, USA; ^8^ Population Sciences Branch, National Heart Lung and Blood Institute, Framingham, MA 01702, USA; ^9^ Psychology Department, University of Haifa, Haifa, Israel; ^10^ Molecular Epidemiology, Leiden University Medical Center, Leiden, Netherlands; ^11^ Max Planck Institute for Biology of Ageing, Köln, Germany; ^12^ Department of Biostatistics, University of Washington, Seattle, WA 98115, USA; ^13^ Rush Alzheimer's Disease Center, Rush University Medical Center, Chicago, IL 60614, USA; ^14^ Translational Gerontology Branch, National Institute on Aging, Baltimore MD 21224, USA; ^15^ Survey Research Center, Institute for Social Research, University of Michigan, Ann Arbor, MI 48104, USA; ^16^ Bioinformatics Core Facility, The Sahlgrenska Academy, University of Gothenburg, Gothenburg, Sweden; ^17^ The University of Texas Health Science Center at Houston, Houston, TX 77030, USA; ^18^ Department of Epidemiology, Erasmus MC, Rotterdam, Netherlands; ^19^ Department of Radiology and Nuclear Medicine, Erasmus MC, Rotterdam, Netherlands; ^20^ Division of Geriatric Medicine, Johns Hopkins Medical Institutes, Baltimore, MD 21224, USA; ^21^ Medicine, Peninsula Health, Peninsula Clinical School, Central Clinical School, Frankston, Melbourne, Victoria, Australia; ^22^ Menzies Institute for Medical Research, University of Tasmania, Hobart, Tasmania, Australia; ^23^ Icelandic Heart Association, Faculty of Medicine, University of Iceland, 101 Reykjavik, Iceland; ^24^ Broad Institute of Harvard and MIT, Cambridge, Harvard Medical School, Department of Neurology, Brigham and Women's Hospital, Boston, MA 02115, USA; ^25^ Rush Institute for Healthy Aging and Department of Internal Medicine, Rush University Medical Center, Chicago, IL 60612, USA; ^26^ Department of Epidemiology, Harvard T.H. Chan School of Public Health, Boston, MA 02115, USA; ^27^ Department of Psychology, University of Edinburgh, Edinburgh, UK; ^28^ Laboratory of Epidemiology and Population Sciences, National Institute on Aging, Intramural Research Program, National Institutes of Health, Bethesda, MD 20892, USA; ^29^ Mayo Clinic, Rochester, MN 55905, USA; ^30^ California Pacific Medical Center Research Institute, San Francisco, CA 94107, USA; ^31^ Division of Nephrology and Hypertension, Mayo Clinic, Rochester, MN 55905, USA; ^32^ Geriatric Unit, Azienda Sanitaria Firenze (ASF), Florence, Italy; ^33^ Gerontology and Geriatrics, Leiden University Medical Center, Leiden, Netherland; ^34^ Centre for Cognitive Ageing and Cognitive Epidemiology, University of Edinburgh, Edinburgh, UK; ^35^ Genetical Statistics, Leiden University Medical Center, Leiden, Netherland. Department of Statistics, University of Leeds, Leeds, UK; ^36^ Department of Internal Medicine and Clinical Nutrition, Institute of Medicine, Sahlgrenska, Academy, University of Gothenburg, Gothenburg, Sweden; ^37^ Department of Neuroscience, Erasmus MC, Rotterdam, Netherlands; ^38^ Clinical and Molecular Osteoporosis Research Unit, Department of Clinical Sciences, Lund University, Malmö, Sweden; ^39^ Laboratory of Neurogenetics, National Institute on Aging, Bethesda, MD 20892, USA; ^40^ Department of Epidemiology and Prevention, Division of Public Health Sciences, Wake Forest University, Winston-Salem, NC 27109, USA; ^41^ School of Computing, Engineering and Mathematics, University of Western Sydney, Sydney, Australia; ^42^ Department of Internal Medicine, Erasmus MC, and Netherlands Genomics Initiative (NGI)-sponsored Netherlands Consortium for Healthy Aging (NCHA), Rotterdam, The Netherlands; ^43^ Alzheimer Scotland Dementia Research Centre, University of Edinburgh, Edinburgh, UK; ^44^ University of Mississippi Medical Center, Jackson, MS 39216, USA; ^45^ Department of Epidemiology, University of Pittsburgh, Pittsburgh, PA 15261, USA; ^46^ Broad Institute of Harvard and MIT, Boston, MA 02131, USA; ^47^ Section of General Internal Medicine, Department of Medicine, Boston University School of Medicine, Boston, MA 02118, USA; ^48^ Department of Human Biology, Faculty of Natural Science, University of Haifa, Haifa, Israel

**Keywords:** gait speed, meta-analysis, aging, GWAS

## Abstract

Emerging evidence suggests that the basis for variation in late-life mobility is attributable, in part, to genetic factors, which may become increasingly important with age. Our objective was to systematically assess the contribution of genetic variation to gait speed in older individuals. We conducted a meta-analysis of gait speed GWASs in 31,478 older adults from 17 cohorts of the CHARGE consortium, and validated our results in 2,588 older adults from 4 independent studies. We followed our initial discoveries with network and eQTL analysis of candidate signals in tissues. The meta-analysis resulted in a list of 536 suggestive genome wide significant SNPs in or near 69 genes. Further interrogation with Pathway Analysis placed gait speed as a polygenic complex trait in five major networks. Subsequent eQTL analysis revealed several SNPs significantly associated with the expression of PRSS16, WDSUB1 and PTPRT, which in addition to the meta-analysis and pathway suggested that genetic effects on gait speed may occur through synaptic function and neuronal development pathways. No genome-wide significant signals for gait speed were identified from this moderately large sample of older adults, suggesting that more refined physical function phenotypes will be needed to identify the genetic basis of gait speed in aging.

## SIGNIFICANCE

Despite promising results from candidate gene studies, a systematic and comprehensive examination of genetic determinants of gait speed in a large sample of older adults has been lacking. Furthermore, previous study samples have been too small to detect the expected modest genetic effects especially in such complex and polygenic encoded traits. To address these limitations, we conducted a meta-analysis of GWAS of gait speed in 31,478 older adults and validate our candidate signal in a cohort of 2588 older adults. Close to 600 candidate genetic variants have been linked to gait speed. Such efforts have provided us with an increased knowledge of the biological systems which impact on gait speed; this may contribute to improved treatment strategies and drug development to promote aging with grace.

## INTRODUCTION

Gait speed has been described as the “sixth vital sign” because it is a core indicator of health and function in aging and disease [1]. Decline in gait speed is ubiquitous with aging in both men and women [2]. Gait speed is used to establish thresholds in community based activities, such as crossing a street [3, 4] or ambulating [5-7]. Slow gait speed is a consistent risk factor for disability, cognitive impairment, institutionalization, falls, hospitalization and mortality [8-10]. Improvement in gait speed is associated with better function and survival.

Many genetic and non-genetic factors (environment and disease) are likely to affect quantitative complex traits such as gait speed. There are individual differences in rates of decline in physical function, and genetic epidemiological studies provide a method for decomposing that variance into genetic and environmental sources. Twin studies suggest that genetic factors account for 15-51% of the variance of gait speed in older adults [11, 12]. Moreover, the contribution of genetic factors may increase with age [2, 11, 13-15]. Offspring of parents with exceptional longevity have better physical function and gait speed in age-specific comparisons to other individuals of comparable age and other characteristics [16, 17]. Effective gait requires the integration of many physiological systems, including the central and peripheral nervous system that create and execute the motor program, the musculoskeletal system that moves and supports the body, and the cardio-pulmonary function that provides perfusion of adequate nutrients and oxygen to all of the integrated parts. All these physiological systems can be affected by genetic variation. Given the many pathways that may contribute to gait impairment, effect sizes of individual genetic variants are expected to be limited.

Previous candidate gene studies have implicated several loci as relevant to gait speed. Single nucleotide polymorphisms (SNP) in the Angiotensin-Converting Enzyme *(ACE)* gene have been linked to better mobility response to exercise. The R577X polymorphism in the alpha-actinin-3 encoding gene (*ACTN3*) was associated with elite athletic performance, and muscle strength and power in the general population, especially in women [18]. There is evidence that *ACE* I/D and *ACTN3* R577X polymorphisms, individually or in combination, have a significant influence on mobility and gait speed phenotypes in older women [19, 20]. Catechol-O-methyltransferase (*COMT*) polymorphisms have been associated with cognitive functions and gait speed [21]. The Met (158) Val polymorphism in *COMT* was linked to faster gait speed in older adults [21]. In addition, apolipoprotein E (*APOE)* genetic variation has been shown to influence the risk of gait speed decline [22-24]. Despite these promising results from candidate gene studies, a systematic and comprehensive examination of genetic determinants of gait speed in a large sample of older adults has been lacking. Furthermore, previous study samples have been too small to detect the expected modest genetic effects [25] especially in such complex and polygenic encoded traits [26].

To address these limitations, we conducted a meta-analysis of GWAS studies of gait speed in 31,478 older adults from the Cohorts for Heart and Aging Research in Genomic Epidemiology (CHARGE) consortium. We then tested our findings in a validation cohort of 2588 older adults participating in four independent studies.

## RESULTS

Gait speed is considered a marker of health and fitness in aging. Slow gait in older adults is associated with increased risk of multiple adverse events including loss of independence, increased risk of disability, falls [27, 28], progression of age-related disease including dementia [29] and death [9]. Slowing of gait is multifactorial with major contributions from potentially modifiable risk factors such as physical inactivity, cognitive impairment, muscle weakness, pain, poor vision, falls and obesity [30]. Gait speed was timed over fixed distance, and reported in m/sec units.

**Figure 1:**
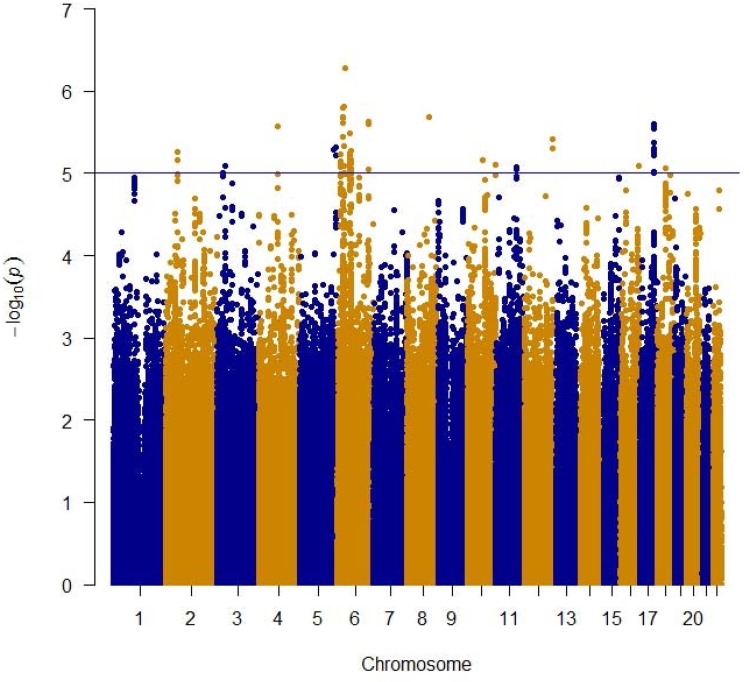
Manhattan plot of meta-analysis of genome wide association studies of gait speed for ∼2.5 million genotype and imputed SNPs The blue line indicates the threshold used to select the 536 suggestive genome wide significant SNPs.

**Figure 2:**
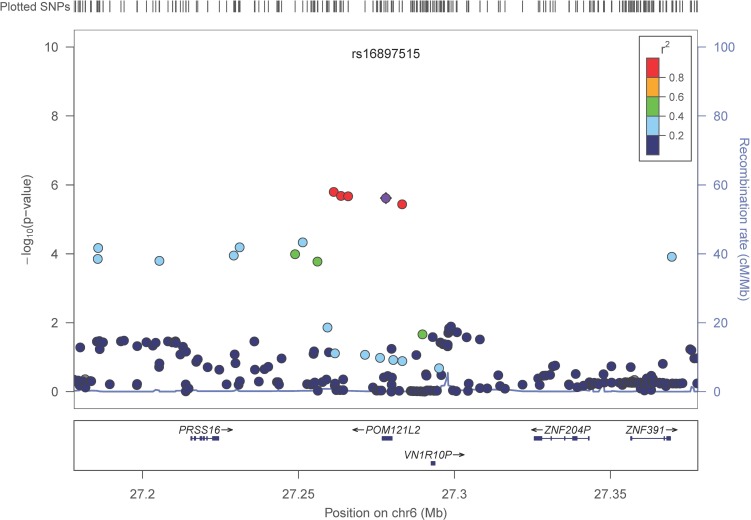

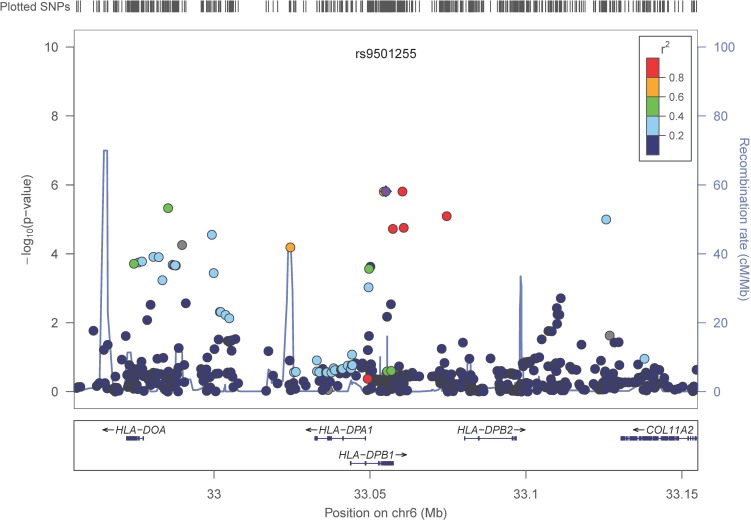

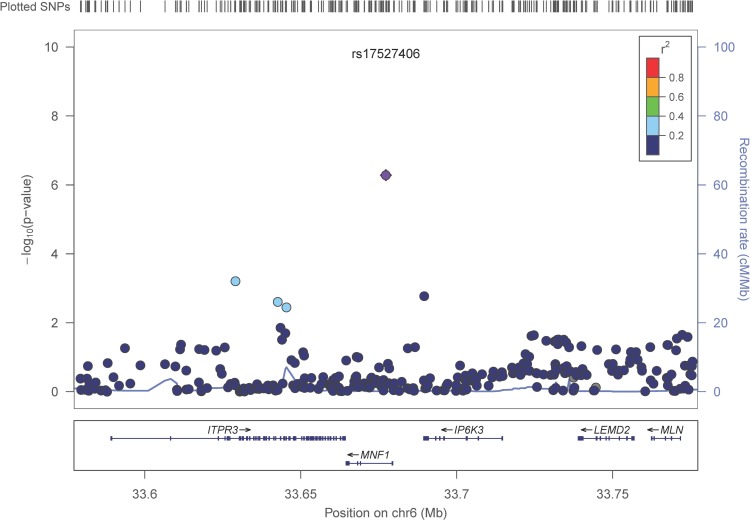

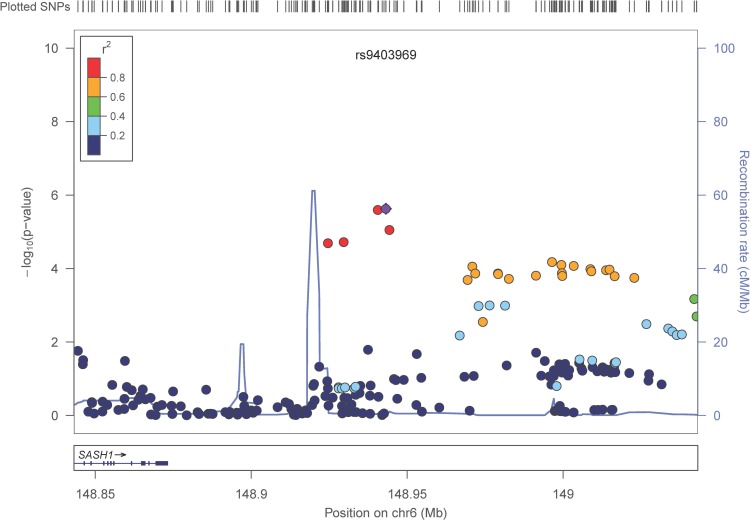

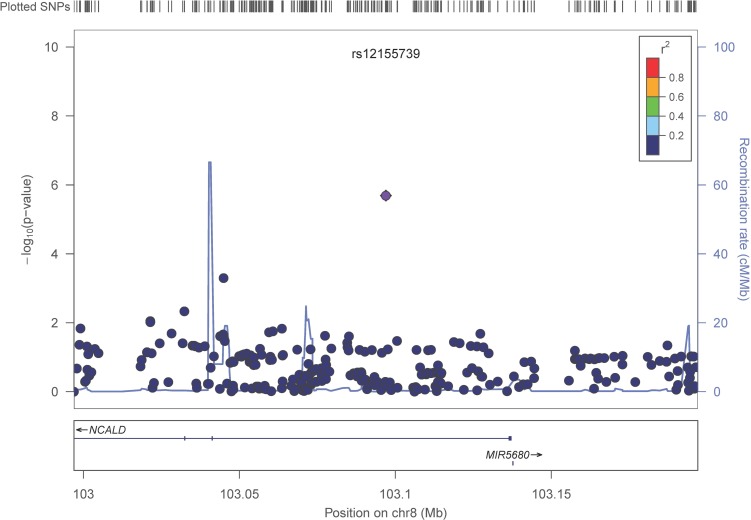
LocusZoom plots for the suggested top 10 SNPs (5 genes) associated with gait speed of the combined analysis (**A**) *POM121L2*; (**B**) *HLA-DPB1*, (**C**) *UQCC2* (MNF1), (**D**) *SASH1*, (**E**) *NCALD*. In each plot, the −log10 of *p* values are on the left *y*-axis; the SNP genomic position (HG19) on the *x*-axis; the estimated recombination rate from 1000 genomes Nov. 2014 EUR are on the right *y*-axis and plotted in blue. The most significant SNP is in purple diamond and plotted using the *p* value attained from the meta-analysis. SNPs are colored to reflect linkage disequilibrium (LD) with the most significant SNP in red (pairwise *r*2 from 1000 genomes Nov. 2014 EUR). Gene annotations are from the SeattleSeqAnnotation141.

In a meta-analysis of 31,478 subjects from 17 cohorts (Table 1, Supplementary Text) with ∼2.5M imputed SNPs (Supplementary Table 1) 536 SNPs (202 were independent (LD, r^2^ < 0.8) based on the HaploReg tool [31]) with p< 1×10^−4^ of which 88 (48 were independent signals) had a p-value less than 1×10^−5^ and one SNP attained a p-value of less than p< 1×10^−6^ (Table 2, Supplementary Table 2). The Q-Q plot (Supplementary Figure 1) did not provide evidence of inflation of test statistics. The Manhattan plot (Figure 1), highlighted 2 regions on chromosome 6 with high LD and suggestive association with gait speed (Regional plots [32] are displayed in Figure 2). These suggestive regions were further interrogated. Although none of the analyzed SNPs were genome wide significant (p< 5×10^−8^), one was present in the top ten *(POM121L2)*, and 7 other genes *(CEP112, PHACTR1, CNTN5*, *PTPRT*, *FHOD3, ADAMTS18, PRIM2*) were highlighted based on the presence of SNPs with suggestive significant associations (p<0.0001) as well as low recombination rate and linkage disequilibrium r^2^ >0.8 which may indicate significant signals in the segment (Figure 2, Supplementary Table 2, Supplementary Figure 2). The 536 suggestive SNPs (p< 1×10^−4^ in the screening group) were tested for validation in four additional cohorts, GENOA, LLS, MrOSGBG and MrOSMalmo (2588 subjects). Among the top 10 SNPs (six independent) only three exceeded nominal significance which slightly improved the combined meta-analysis significance for *HLA-DPB1* SNPs (rs9501255, rs7763822 & rs3749985), however genome-wide levels of signifi-cance were not attained (Table 2).

**Table 1:** Demography of the screening and validation cohorts

	Cohort	Age, y	%Female	N with gait and GWAS	Gait protocol
Screening	AGES	>65	58.9	3,166	6 meter walk
	ARIC	>60	59.5	445	7.6 meter walk
	BLSA	>60	49.5	334	6 meter walk
	CHS	≥65	60.9	3,184	4.6 meter walk
	FHS	>65	56.1	2,384	4 meter walk
	HABC	>70	47.1	1,482	6 meter walk
	HRS	>65	56.4	5,073	2.5 meter walk
	InCHIANTI	> 60	55.8	898	4 meter walk
	LBC1921	77-80	58.4	510	6 meter walk
	LBC1936	67-71	49.5	1,001	6 meter walk
	MrOS	≥65	None	4,643	6 meter walk
	ROSMAP	>60	69.2	1,646	2.5 meter walk
	RS-I	>55	53	706	6 meter walk
	RS-II	>55	51.8	813	6 meter walk
	RS-III	>45	56.0	1,392	6 meter walk
	SOF	≥65	100	3,441	6 meter walk
	TASCOG	>60	42	360	6 meter walk
Total Screening				31,478	
Validation	GENOA	>60	55	471	7.6 meter walk
	LLS	>60	47.2	235	4 meter walk
	MrOSGBG	>69	None	960	6 meter walk
	MrOSMalmo	>69	None	922	6 meter walk
Total Validation				2,588	

**Table 2:** Top 10 association meta-analysis results for gait speed

						Screening Set (n=31,478)			Validation Set (n=2,588)		Screening + Validation Set (n=34,066)	
SNP	Chr.:Position	E/NE Allele	F E Allele	Closest Gene	Δ(kb)/gene location	Beta (SE)	P	HetPVal	Beta (SE)	P	Beta (SE)	P
rs17527406	6:33709545	C/G	0.016	*UQCC2(MNF1)*	intron	0.040(0.007)	5.22E-7	0.2669	0.014(0.032)	0.65	0.037(0.007)	6.883e-7
rs9501255*	6:33087321	T/C	0.038	*HLA-DPB1*	3′ UTR	0.023(0.005)	1.53e-6	0.5853	0.048(0.023)	0.04	0.024(0.005)	3.326e-7
rs7763822*	6:33092651	T/C	0.038	*HLA-DPB1*	3	0.023(0.005)	1.54e-6	0.5704	0.047(0.023)	0.04	0.024(0.005)	3.440e-7
rs3749985*	6:33086656	C/G	0.038	*HLA-DPB1*	3′ UTR	0.023(0.005)	1.55e-6	0.5856	0.048(0.023)	0.04	0.024(0.005)	3.385e-7
rs7746199#	6:27293545	C/T	0.166	*POM121L2*	15	0.011(0.002)	1.58E-6	0.9658	0.011(0.008)	0.19	0.011(0.002)	7.125e-7
rs12155739	8:102084750	C/T	0.030	*NCALD*	intron	−0.041(0.008)	2.04E-6	0.2076	−0.024(0.032)	0.45	−0.039(0.008)	1.858e-6
rs3800318#	6:27295862	A/T	0.830	*POM121L2*	13	−0.011(0.002)	2.07E-6	0.966	−0.011(0.008)	0.20	−0.011(0.002)	9.686e-7
rs13211166	6:27298161	A/T	0.190	*POM121L2*	11	0.011(0.002)	2.12E-6	0.9688	0.010(0.009)	0.24	0.010(0.002)	1.136e-6
rs9403969	6:148622038	T/G	0.737	*SASH1*	70	0.009(0.002)	2.34e-6	0.4004	0.005(0.007)	0.50	0.009(0.002)	2.351e-6
rs16897515#	6:27310241	A/C	0.161	*POM121L2*	missense	0.011(0.002)	2.41E-6	0.9505	0.007(0.009)	0.42	0.010(0.002)	2.080e-6

*First gene segment, #second gene segment. E/NE-Effect-, Non-Effect allele; F E-Frequency of Effect Allele; Δ-distance to proximal gene; HetPVal- Heterogeneity P Value.

### Candidate gene approach

None of the imputed variants previously reported as gait speed candidate genes such as *ACE*, *ACTN3, COMT and APOE* reached a nominally significant (p<0.05) threshold (Supplementary Table 3).

### Pathway analysis

**Figure 3:**
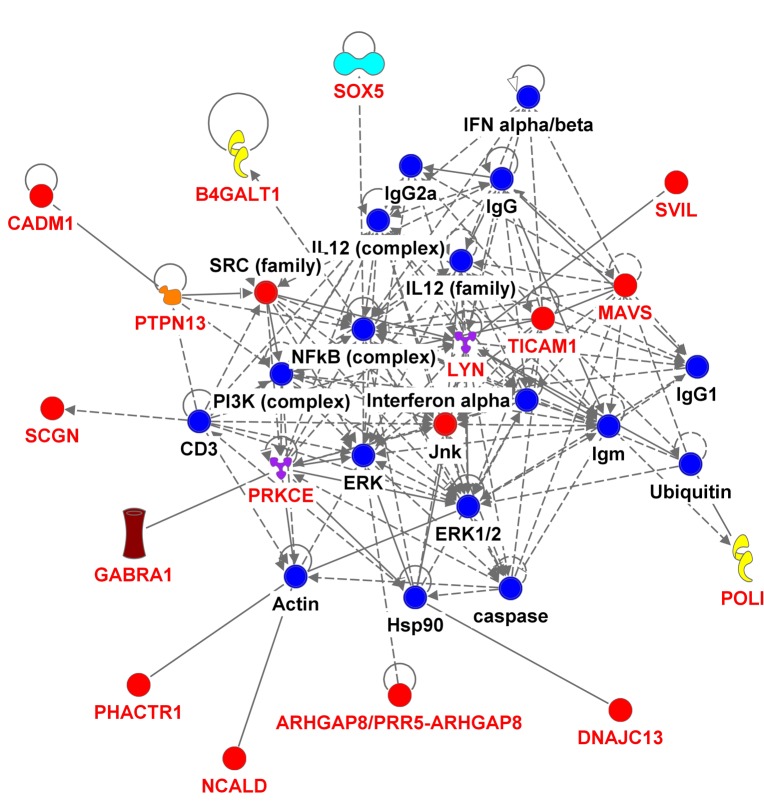

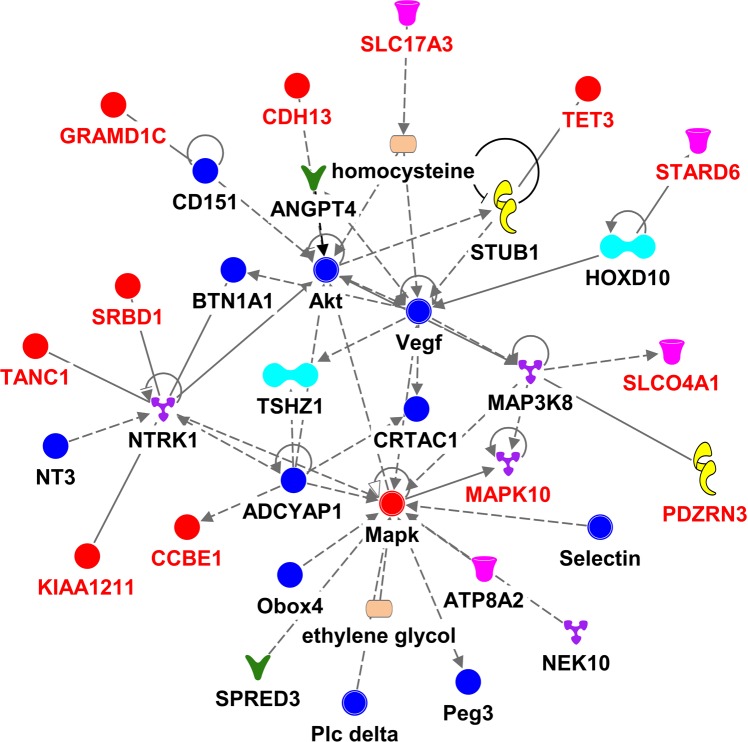

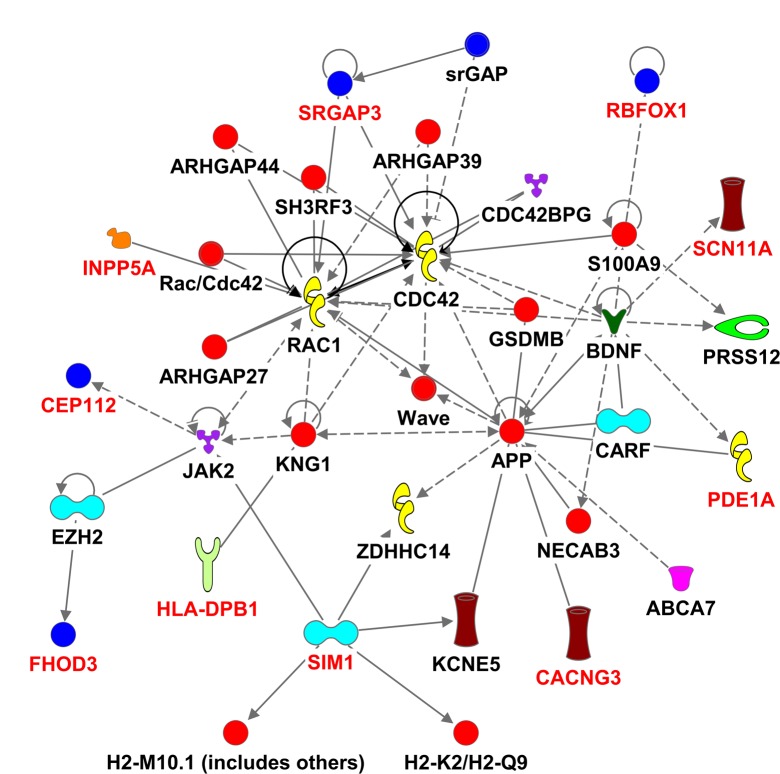

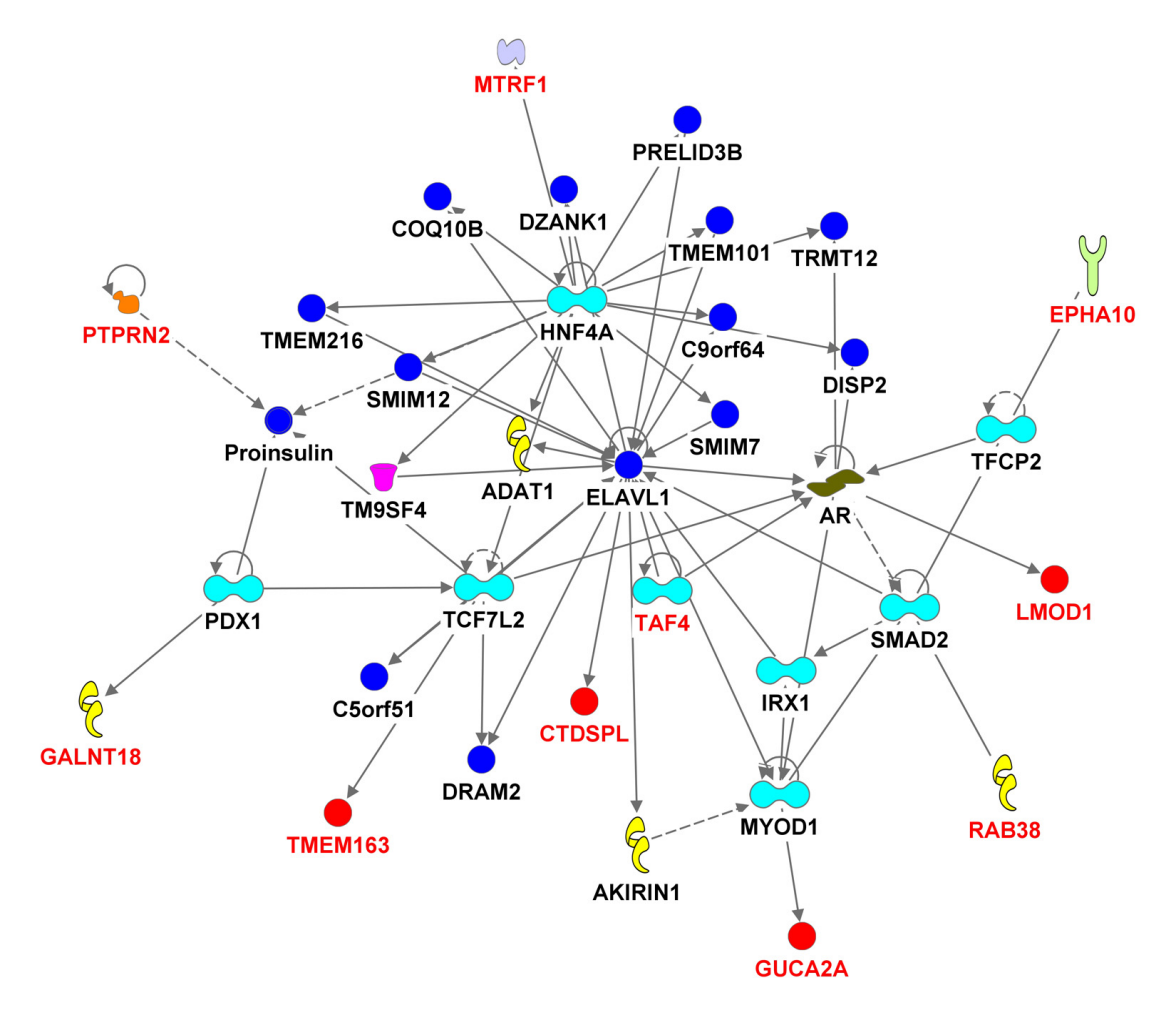

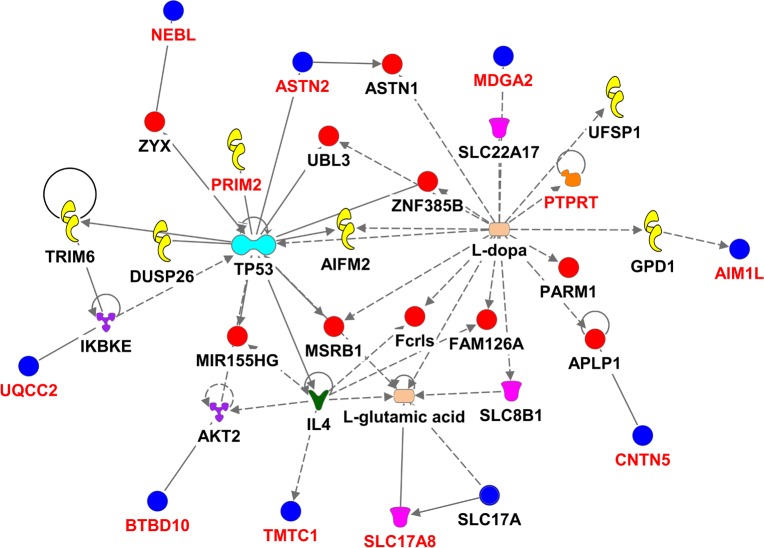
Ingenuity pathway analysis of genes associated with gait speed Genes are represented as nodes; solid lines indicate direct- and hatched lines indirect- interaction. Gene functions are color-coded as follows: Red= other, Navy Blue =Group/Complex, Yellow= Enzyme, Turquoise= transcription regulator, Brown= Ion Channel, Orange= Phosphatase, Purple = Kinase, Magenta= Transporter, Beige=chemical-endogenous mammalian, Hunter Green (Dark Green) = Growth factor, light Green= Transmembrane Receptor, Light Purple= Translation Regulator, Olive Green=Ligand-dependent nuclear receptor, Bright green= Peptidase.

We used the 536 suggestive SNPs to generate the network analysis, in which 283 SNPs representing 68 genes (Supplementary Table 4) were located in both the IPA dataset and the SeattleSeqAnnotation141 for SNP annotation (the remaining 253SNPs did not map to a gene). Among the genes having the highest number of defining SNPs, were *CEP112* (38 SNPs), *PHACTR1* (23 SNPs), *CNTN5* (19 SNPs), *PTPRT* (18 SNPs), *FHOD3* (17 SNPs), *ADAMTS18* (12 SNPs) and *PRIM2* (11 SNPs). The vast majority of these genes’ products are located in the cytoplasm and plasma membrane while the rest are in the nucleus, extracellular space and other cellular spaces. Ten types of protein actions (enzyme, transporter, phosphatase, transcription regulator, kinase, ion channel, transmembrane receptor, translation regulator, ligand- dependent nuclear receptor and peptidase) are enumerated in Supplementary Table 5. Five of them serve as a biomarker for diagnosis, disease progression, prognosis, and unspecified application and five of them were targets for drug development including *PRIM2, GABRA1, LYN, PRKCE* and *SCN11A*. Five major putative disease and function networks were established using the candidate genes (based on the IPA software analysis significance classification) and were classified accordingly to cancer, gastrointestinal disease, organismal injury and abnormalities, neurological disease, cell and tissue morphology, cellular function, development and maintenance, amino acid metabolism, small molecule biochemistry, gene expression, cell-to-cell signaling and interaction, nervous system development and function, cellular assembly and organization. Seven genes were not mapped to any network (see Figure 3, Supplementary Table 6).

### eQTL analysis

By querying a large collection of eQTL results (listed in Supplementary Text), we obtained a long list of possible SNP relationships with gene expression (Supplementary Table 7). We also identified the strongest eQTL SNP for each particular transcript in each study. Those SNPs with low p-values (for association with gene expression, p<10^−8^) and high LD (D'>0.9) with the functional variant, were picked as candidates of signal concordance between the eQTL signals and gait speed signal. Following this analysis, several transcripts including PRSS16 and WDSUB1 were highlighted (Supplementary Table 7). We also observed a relationship between a SNP and PTPRT expression (in liver tissue), which in addition to the meta-analysis and pathway analysis emphasized its potential functional link through its synaptic function and neuronal development, both of which may contribute to [33] gait speed. By emphasizing a strong relationship of the best eQTL with our queried SNPs, we likely underreport SNP-expression relationships due to missing LD information and the inability to project LD relationships for trans-eQTLs in the region.

Applying HaploReg v4.1 analysis to the 536 variants resulted in 9 categories (Supplementary Table 8): miscRNA (1 variant); snoRNA (2 variants); microRNA (4 variants); snRNA (9 variants); pseudogenes (14 variants); sequencing in progress (43 variants); LINC RNA (86 variants); and 372 variants within protein coding genes. In addition, some variants annotate to the same gene resulting in a total of 139 genes (protein-coding or non-coding). Of those genes, 6 are exceptionally long, containing over a million base-pairs, the longest of which is PTPRT coded by 1117219bp. The shortest genes are the ones coding for micro (MIR3143) or small nuclear (U7) RNAs at 63bp each. There is only partial information regarding the chromatin state of each variant. However, from the information gathered in the analysis we observed 14 transcription start sites and 245 enhancers (Supplementary Table 8).

## DISCUSSION

In this genome-wide association study of gait speed in 31,478 adults ages 60 and older from 17 different cohorts in the USA, Europe and Australia and 2,588 individuals in four validation cohorts, we did not discover any genome-wide significant association with gait speed nor did we confirm gait speed associations with previously reported candidate genes (i.e. *ACE*, *ACTN3, COMT and APOE)* (Supplementary Table 3). However, our analyses revealed some potentially relevant SNPs that could be targeted for further analyses regarding their associations with gait speed.

Our results shed light on several candidate genetic polymorphisms that did not achieve genome wide significance but which had multiple signals on the gene segment, an observation that supported the association with the trait of interest. In addition, these SNPs map to genes that were either linked to physiologic functions expected to influence gait speed (such as neuromuscular function, cardiac function and muscle health or brain function) *ADAMTS18,* a gene associated with bone mineral density, could be associated with gait speed if individuals with variants in this gene had suffered from fracture leading to slowing of gait [34]. In functional studies *ADAMTS18* levels were significantly lower in subjects with non-healing skeletal fractures compared to normal subjects [35]. *POM121L2* - an ion transport gene [36] - was listed in the top ten meta-analysis genes with four variants, making it a potential candidate for our study. This gene has been linked to schizophrenia, [37], suggesting a potential brain-related association with gait speed. One of the top candidates in our analysis was *UQCC2* (also known as M19 or MNF1), a mitochondrial membrane protein that regulates skeletal muscle differentiation and insulin secretion [38]. Although *UQCC2* function has a clear link to gait speed, the fact that in this study only one SNP found within *UQCC2* demonstrated suggestive significance, which provides less confidence of a true association. *NCALD*, a calcium-binding protein, has been associated with diabetic nephropathy [39]. The region that was highlighted next to *SASH1*, a tumor suppressor gene, has multiple signals associated with gait speed. However, there is a high recombination rate between this region and the candidate gene (Figure 2), suggesting a higher dissociation between the gene and the signaled region. The last candidate from the top 10 SNP association list is *HLA-DPB1*, an immune response gene that has been linked to rheumatoid and inflammatory myopathies [40, 41]. Interestingly, one of its variants (rs7763822) was indicated in systemic sclerosis susceptibility in Korean subjects [42] suggesting a pleiotropic effect.

*CEP112* involved in proper cell cycle progression [43] was not listed among the top 10 SNPs (Table 2) however its clear dominancy (38 SNPs) among the 536 suggestive SNPs make it an attractive candidate for further functional association studies with gait speed. Similar to *CEP112* variants, *PHACTR1* regulates cardiac α-actin isoform ratio [44] and actomyosin assembly [45]; *CNTN5* is associated with neuron function [46]; *PTPRT* regulates synaptic function and neuronal development [33] and serves as a genuine susceptibility locus for rheumatoid arthritis[33]; *FHOD3*, is a key regulator in the cardiac muscle [47] and sarcomere organization in striated muscle cells [48]; and PRIM2 is involved in DNA replication and transcription and is crucial for normal growth and development [49]. This list of genes repeatedly implicates associated signals that are important for neuromuscular function, cardiac function and muscle health, which could reasonably contribute to the complex trait of gait speed.

A second tier of locus with repetitive signals established among the 536 suggestive SNPs included *PDZN3*, which is implicated in muscle function and regeneration [50-52], *CACNG3*, a voltage-dependent calcium channel subunit [53] that was previously linked to ataxic phenotype in mice [54], *ASTN2* that functions in neuronal migration [55] and that was associated with hip osteoarthritis susceptibility [56], *SIM1* involved in coordinating muscle activity and generating rhythmic activity [57] and also associated with obesity [58], and *MDGA2*, which is required for proper development of cranial motoneuron subtypes [59].

The eQTL analysis (various tissues and cell types, listed in Supplementary Text) of the 536 suggestive SNPs reported a couple of candidate genes such as *PRSS16*, a gene encoding serine protease expressed exclusively in the thymus. *PRSS16* was associated with exercise [60] and was linked to *COMT* (a candidate gene for gait speed *(20)*). Both are regulated by *ZNF804a* [61]. This link between the two genes (*PRSS16* and *COMT*) may support our gait speed association results. Another candidate gene from our eQTL analysis was *WDSUB1* a U-box ubiquitin ligases encoded protein which was associated with sudden cardiac death [62]. A link with cardiovascular diseases may indicate a potential cardiovascular effect on gait speed. The last candidate in this analysis is *PTPRT*, a gene that regulates synaptic function and neuronal development. It is possible that its link to gait speed (operates through its role in diabetes [63]). The fact that it was present in all three sets of analysis results may suggest a stronger candidate for further analysis.

The lead motif of the network analysis in all 5 disease networks was “cellular function”, however, the candidate SNPs from the multiple analysis strategies strongly suggested links to bone, skeleton, muscle and brain, incorporating development, structure and function. While our SNP associations did not achieve genome wide significance, we believe that we demonstrated a potential link to gait speed. To exclude false positive signals, these associations should be pursued further in controlled experiments as well as animal models, which will increase our understanding of the biology of gait speed deterioration with aging. Such efforts would provide us with an increased knowledge of the biological systems which impact on gait speed; this may contribute to improved treatment strategies and drug development to promote aging with grace.

This study did not provide conclusive evidence for the genetics contributing to gait speed. While the large sample is a strength (and we have the power to detect smaller effects), the observed associations suggest that an even larger sample is required to establish genetic contributions to the gait speed phenotype. The individual effects of common SNPs for complex traits such as gait speed are expected to be very small. From studies of other polygenic complex traits, it has been observed that the number of discovered variants is strongly correlated with experimental sample size [64]. Another potential explanation why we did not observe genome wide significant associations is that there are many potential pathways that contribute to gait speed, including nervous system function (neuromuscular, central nervous system), musculoskeletal conditions such as sarcopenia and osteoarthritis, cardiovascular disease, visual function, psychological factors and other contributors. This complexity of phenotype may make it difficult to discover associations. Phenotype refinement may be a future approach to explore.

In summary, the lack of genome-wide significant signals from this moderately large sample of older adults suggests that larger samples (or study to sub-classify the gait speed phenotype) will be needed to identify SNP-based associations. Also, it may suggest that downstream mechanisms are more likely to make more important contributions to gait speed. Gait speed is a complex phenotype with many potential contributors; it is not unsurprising that it should be governed by multiple genes. However, we were able to use network analyses to define some potential networks of genes that might be of relevance for this phenotype. Future studies may be best positioned to focus on one network in more detail and to examine gene-environment or gene-behavior- environment interactions.

## METHODS

### Subjects

The Aging and Longevity Working Group of the CHARGE Consortium [65, 66], was formed to facilitate genome-wide association study meta-analyses of age associated diseases and phenotypes among multiple large and well-phenotyped cohorts of older individuals who underwent genotyping.

#### Screening cohorts

A combined cohort of 31,478 subjects age 60 years and older with timed walks constituted our discovery sample (Table 1). Timed walk at usual pace was converted to gait speed (m/s) to harmonize the phenotype across cohorts. Participants of the following 17 European descendent cohorts were included (Supplementary Material):

The Age, Gene/Environment Susceptibility-Reykjavik (AGES), The Atherosclerosis Risk in Communities (ARIC), Baltimore Longitudinal study on Aging (BLSA), Cardiovascular Health Study (CHS), Framingham Heart Study (FHS), Health, Aging, and Body Composition Study (HABC), Health and Retirement Study (HRS), Invecchiare in Chianti (InCHIANTI), Lothian Birth Cohorts 1921 (LBC1921) and 1936 (LBC1936), Osteoporotic Fractures in Men Study (MrOS), The Religious Orders Study and Rush Memory and Aging Project (ROSMAP), Rotterdam Study (RS-I, -II, -III), Study of Osteoporotic Fractures (SOF), Tasmanian Study of Cognition and Gait (TASCOG) (Table 1, Supplementary Text). All participants with gait speed assessments including participants who were able to walk with assistance of a cane were included in this analysis. Exclusion criteria included missing gait assessments and inability to walk (Supplement Text).

#### Validation cohorts

The validation cohort consisted of 2,588 subjects (>60 years) from the Genetic Epidemiology Network of Arteriopathy (GENOA), Leiden Longevity Study (LLS), Osteoporotic Fractures in Men Study (MrOS) Sweden, Malmö[MrOSMalmo] and Gothenburg [MrOSGBG] studies (Table 1, Supplement text). Together these cohorts reach the minimum number of subjects required for sufficient statistical power (Our power calculation shows that given a fixed sample size (n=2500) our analysis will have >80% power to detect MAF=0.01, alpha<0.0001) to validate significant signal(s) from the screening cohort using the same harmonized gait speed phenotype. Results from the screening and validation cohorts were meta-analyzed.

### Phenotype definition

The different methods of assessing gait speed in individual cohorts are described in Table 1 and Supplementary Text. Variability in the methods of assessing gait speed in the participating cohorts included differences in distance walked (8 to 25 feet) and measurement techniques (instrumented walkway versus stopwatch). Previous reports including 4 cohorts from this report (CHS, HABC, InCHIANTI and SOF) have suggested that there is a high correlation (r^2^>0.9) between the different methods of measuring gait speed [9, 24, 67]. The mean overall gait speed was 1.13± 0.25 m/sec, and varied from 0.66 ± 0.16 m/sec to 1.66 ± 0.41 m/sec in the individual cohorts (Supplementary Table 9, Supplementary Figure 3).

### Genotyping

A structured, pre-specified analytical plan was applied to each of the 17 cohorts included in the screening sample. Genome-wide analysis of imputed genotypes, summarized in Supplementary Text, were conducted in each cohort. Imputation (using either BimBam or MACH) resulted in approximately 2.5 million HapMap SNPs being available for analysis. Imputation details, QC and SNP count per cohort can be found in Supplementary Text and Supplementary Table 1. Exclusion criteria for SNP in each of the 21 cohorts (screening and validation) included: 1) minor allele frequency (MAF) < 0.005); 2) imputation quality (R^2^ or oevar_imp < 0.3); and for the meta-analysis, SNPs with average MAF ≤ 0.01 and total N < 15,000.

### Cohort-specific analyses

Multiple linear regression of imputed SNP dosages on gait speed was performed using an additive model, i.e. as a count of the number of variant alleles present (1 degree of freedom). Sex-combined analysis was performed. Adjustment for age (at time of exam), sex, study site (for cohorts with multiple sites), principal components to control for population stratification, height, and presence of osteoarthritis (yes/no) if available were included. For cohorts with osteoarthritis data available, the analysis was done excluding participants with osteoarthritis (Supplementary Text and Supplementary Table 10).

### Meta-analysis

Inverse variance weighted meta-analysis was performed on summary statistics of the cohort-level association analyses. Meta-analysis of gait speed (Screening and validation cohorts were analyzed separately as well as together (joint meta-analysis)) was performed using METAL [68] with a fixed effects model of beta estimates and standard errors from each cohort. In addition, we applied heterogeneity test between studies (on both screening and validation cohorts) using METAL. A p-value threshold (Bonferroni-adjusted) of p<5×10^−8^ was used to indicate genome-wide statistical significance.

### Pathway analysis

We assembled a list of 536 meta-analyzed SNPs (representing 69 genes) that were highly suggestively associated (p < 1 × 10^−4^) with gait speed. This list resulted in 67 candidate genes (Annotated by Ingenuity Pathway Analysis (IPA) and SeattleSeqAnnotation) being identified which were used in the IPA analysis (www.ingenuity.com). The resulting classification of networks, pathways, biological processes and molecular functions are represented in tables and graphic format (Figure 3, Supplementary Table 4, 5 and 6).

### Expression quantitative trait loci (eQTL) analysis

We examined existing eQTL resources for the candidate suggestive list of 536 SNPs (p<10^−4^) to further explore their biological and functional relevance to gait speed (Supplementary Text). We queried these SNPs against an eQTL database (listed in Supplementary Text) containing eQTL results from over 100 studies across a wide range of tissues. A general overview of a subset of >50 eQTL studies has been published [69], with specific citations for the included datasets included in the Supplementary Material.

Further we applied the HaploReg v4.1 annotation tool for TF analysis of 536 SNPs suggestively associated with gait speed.

## SUPPLEMENTARY MATERIAL FIGURES AND TABLES
























