# Correction: The complex genetics of gait speed: genome-wide meta-analysis approach

**DOI:** 10.18632/aging.101260

**Published:** 2017-07-07

**Authors:** Dan Ben-Avraham, David Karasik, Joe Verghese, Kathryn L. Lunetta, Jennifer A. Smith, John D. Eicher, Rotem Vered, Joris Deelen, Alice M. Arnold, Aron S. Buchman, Toshiko Tanaka, Jessica D. Faul, Maria Nethander, Myriam Fornage, Hieab H. Adams, Amy M. Matteini, Michele L. Callisaya, Albert V. Smith, Lei Yu, Philip L. De Jager, Denis A. Evans, Vilmundur Gudnason, Albert Hofman, Alison Pattie, Janie Corley, Lenore J. Launer, Davis S. Knopman, Neeta Parimi, Stephen T. Turner, Stefania Bandinelli, Marian Beekman, Danielle Gutman, Lital Sharvit, Simon P. Mooijaart, David C. Liewald, Jeanine J. Houwing-Duistermaat, Claes Ohlsson, Matthijs Moed, Vincent J. Verlinden, Dan Mellström, Jos N. van der Geest, Magnus Karlsson, Dena Hernandez, Rebekah McWhirter, Yongmei Liu, Russell Thomson, Gregory J. Tranah, Andre G. Uitterlinden, David R. Weir, Wei Zhao, John M. Starr, Andrew D. Johnson, M. Arfan Ikram, David A. Bennett, Steven R. Cummings, Ian J. Deary, Tamara B. Harris, Sharon L. R. Kardia, Thomas H. Mosley, Velandai K. Srikanth, Beverly G. Windham, Ann B. Newman, Jeremy D. Walston, Gail Davies, Daniel S. Evans, Eline P. Slagboom, Luigi Ferrucci, Douglas P. Kiel, Joanne M. Murabito, Gil Atzmon

**Affiliations:** ^1^ Department of Medicine and Genetics Albert Einstein College of Medicine, Bronx, NY 10461, USA; ^2^ Institute for Aging Research, Hebrew SeniorLife, Department of Medicine, Beth Israel Deaconess Medical Center and Harvard Medical School, Boston, MA 02131, USA; ^3^ Faculty of Medicine in the Galilee, Bar-Ilan University, Safed, Israel; ^4^ Integrated Divisions of Cognitive & Motor Aging (Neurology) and Geriatrics (Medicine), Montefiore-Einstein Center for the Aging Brain, Albert Einstein College of Medicine, Bronx, NY 10461, USA; ^5^ The National Heart Lung and Blood Institute's Framingham Heart Study, Framingham, MA 01702, USA; ^6^ Department of Biostatistics, Boston University School of Public Health, Boston, MA 02118, USA; ^7^ Department of Epidemiology, School of Public Health, University of Michigan, Ann Arbor, MI 48109, USA; ^8^ Population Sciences Branch, National Heart Lung and Blood Institute, Framingham, MA 01702, USA; ^9^ Psychology Department, University of Haifa, Haifa, Israel; ^10^ Molecular Epidemiology, Leiden University Medical Center, Leiden, Netherlands; ^11^ Max Planck Institute for Biology of Ageing, Köln, Germany; ^12^ Department of Biostatistics, University of Washington, Seattle, WA 98115, USA; ^13^ Rush Alzheimer's Disease Center, Rush University Medical Center, Chicago, IL 60614, USA; ^14^ Translational Gerontology Branch, National Institute on Aging, Baltimore MD 21224, USA; ^15^ Survey Research Center, Institute for Social Research, University of Michigan, Ann Arbor, MI 48104, USA; ^16^ Bioinformatics Core Facility, The Sahlgrenska Academy, University of Gothenburg, Gothenburg, Sweden; ^17^ The University of Texas Health Science Center at Houston, Houston, TX 77030, USA; ^18^ Department of Epidemiology, Erasmus MC, Rotterdam, Netherlands; ^19^ Department of Radiology and Nuclear Medicine, Erasmus MC, Rotterdam, Netherlands; ^20^ Division of Geriatric Medicine, Johns Hopkins Medical Institutes, Baltimore, MD 21224, USA; ^21^ Medicine, Peninsula Health, Peninsula Clinical School, Central Clinical School, Frankston, Melbourne, Victoria, Australia; ^22^ Menzies Institute for Medical Research, University of Tasmania, Hobart, Tasmania, Australia; ^23^ Icelandic Heart Association, Faculty of Medicine, University of Iceland, 101 Reykjavik, Iceland; ^24^ Broad Institute of Harvard and MIT, Cambridge, Harvard Medical School, Department of Neurology, Brigham and Women's Hospital, Boston, MA 02115, USA; ^25^ Rush Institute for Healthy Aging and Department of Internal Medicine, Rush University Medical Center, Chicago, IL 60612, USA; ^26^ Department of Epidemiology, Harvard T.H. Chan School of Public Health, Boston, MA 02115, USA; ^27^ Department of Psychology, University of Edinburgh, Edinburgh, UK; ^28^ Laboratory of Epidemiology and Population Sciences, National Institute on Aging, Intramural Research Program, National Institutes of Health, Bethesda, MD 20892, USA; ^29^ Mayo Clinic, Rochester, MN 55905, USA; ^30^ California Pacific Medical Center Research Institute, San Francisco, CA 94107, USA; ^31^ Division of Nephrology and Hypertension, Mayo Clinic, Rochester, MN 55905, USA; ^32^ Geriatric Unit, Azienda Sanitaria Firenze (ASF), Florence, Italy; ^33^ Gerontology and Geriatrics, Leiden University Medical Center, Leiden, Netherland; ^34^ Centre for Cognitive Ageing and Cognitive Epidemiology, University of Edinburgh, Edinburgh, UK; ^35^ Genetical Statistics, Leiden University Medical Center, Leiden, Netherland. Department of Statistics, University of Leeds, Leeds, UK; ^36^ Department of Internal Medicine and Clinical Nutrition, Institute of Medicine, Sahlgrenska, Academy, University of Gothenburg, Gothenburg, Sweden; ^37^ Department of Neuroscience, Erasmus MC, Rotterdam, Netherlands; ^38^ Clinical and Molecular Osteoporosis Research Unit, Department of Clinical Sciences, Lund University, Malmö, Sweden; ^39^ Laboratory of Neurogenetics, National Institute on Aging, Bethesda, MD 20892, USA; ^40^ Department of Epidemiology and Prevention, Division of Public Health Sciences, Wake Forest University, Winston-Salem, NC 27109, USA; ^41^ School of Computing, Engineering and Mathematics, University of Western Sydney, Sydney, Australia; ^42^ Department of Internal Medicine, Erasmus MC, and Netherlands Genomics Initiative (NGI)-sponsored Netherlands Consortium for Healthy Aging (NCHA), Rotterdam, The Netherlands; ^43^ Alzheimer Scotland Dementia Research Centre, University of Edinburgh, Edinburgh, UK; ^44^ University of Mississippi Medical Center, Jackson, MS 39216, USA; ^45^ Department of Epidemiology, University of Pittsburgh, Pittsburgh, PA 15261, USA; ^46^ Broad Institute of Harvard and MIT, Boston, MA 02131, USA; ^47^ Section of General Internal Medicine, Department of Medicine, Boston University School of Medicine, Boston, MA 02118, USA; ^48^ Department of Human Biology, Faculty of Natural Science, University of Haifa, Haifa, Israel

Original article: Aging (Albany NY) 2017; 9(1): 209-246.

PMCID: PMC 5310665 PMID: 28077804 DOI: 10.18632/aging.101151

Please check the link to the original paper: http://www.aging-us.com/article/101151/text

**Present**: Due to proofreading oversight, there is a mistake (marked in bold) in the last paragraph before DISCUSSION on page 219

Applying HaploReg v4.1 analysis to the 536 variants resulted in 9 categories (Supplementary Table 8): miscRNA (1 variant); snoRNA (2 variants); microRNA (4 variants); snRNA (9 variants); pseudogenes (14 variants); sequencing in progress (43 variants); LINC RNA (86 variants); and 372 variants within protein coding genes. In addition, some variants annotate to the same gene resulting in a total of 139 genes (protein-coding or non-coding). Of those genes, 6 are exceptionally long, containing over a million base-pairs, the longest of which is **PTPRD coded by 2298477bp**. The shortest genes are the ones coding for micro (MIR3143) or small nuclear (U7) RNAs at 63bp each. There is only partial information regarding the chromatin state of each variant. However, from the information gathered in the analysis we observed 14 transcription start sites and 245 enhancers (Supplementary Table 8).

**Corrected**: The corrected text is provided below.

Applying HaploReg v4.1 analysis to the 536 variants resulted in 9 categories (Supplementary Table 8): miscRNA (1 variant); snoRNA (2 variants); microRNA (4 variants); snRNA (9 variants); pseudogenes (14 variants); sequencing in progress (43 variants); LINC RNA (86 variants); and 372 variants within protein coding genes. In addition, some variants annotate to the same gene resulting in a total of 139 genes (protein-coding or non-coding). Of those genes, 6 are exceptionally long, containing over a million base-pairs, the longest of which is **PTPRT coded by1117219bp**. The shortest genes are the ones coding for micro (MIR3143) or small nuclear (U7) RNAs at 63bp each. There is only partial information regarding the chromatin state of each variant. However, from the information gathered in the analysis we observed 14 transcription start sites and 245 enhancers (Supplementary Table 8).

The authors sincerely apologize for this error.

