# Supplementary material for: The complex genetics of gait speed: genome-wide meta-analysis approach
Source: Aging (Albany NY). 2017 Jan 10;9(1):209–46. doi: 10.18632/aging.101151 (PMC5310665; doi:10.18632/aging.101151)
Supplement: Supplementary file 1 [file aging-09-0209-s001.pdf]

SUPPLEMENTARY MATERIAL

SUPPLEMENTARY TEXT

Cohort description

The Age, Gene/Environment Susceptibility-Reykjavik (AGES)

The Reykjavik Study cohort originally comprised a random sample of 30,795 men and women born in 1907–1935 and living in Reykjavik in 1967 [1]. A total of 19381 attended, resulting in 71% recruitment rate. The study sample was divided into six groups by birth year and birth date within month. One group was designated for longitudinal follow-up and was examined in all stages. One group was designated a control group and was not included in examinations until 1991. Other groups were invited to participate in specific stages of the study. Between 2002 and 2006, the AGES-Reykjavik study re-examined 5764 survivors of the original cohort who had participated before in the Reykjavik Study. Of those, 3,219 have genomic genotypes and only 3,166 went through gait assessment that included 6 meter walk in usual pace.

The Atherosclerosis Risk in Communities (ARIC)

The ARIC study is a population-based cohort study of atherosclerosis and clinical atherosclerotic diseases [2]. At its inception (1987-1989), 15,792 men and women, including 11,478 white and 4,266 black participants were recruited from four U.S. communities: Suburban Minneapolis, Minnesota; Washington County, Maryland; Forsyth County, North Carolina; and Jackson, Mississippi. In the first 3 communities, the sample reflects the demographic composition of the community. In Jackson, only black residents were enrolled. Participants were between age 45 and 64 years at their baseline examination in 1987-1989 when blood was drawn for DNA extraction and participants consented to genetic testing. Between 2004 and 2006, participants who had undergone magnetic resonance scanning at the third ARIC visit were invited to participate in the ARIC MRI study [3]. Gait assessment was performed on 1134 ARIC participants. Time to walk 25 feet (7.62 m) at the participants' usual pace was recorded in an unobstructed corridor with a stop watch. Four hundred and forty five participants of European ancestry with genome-wide genotype data and a gait speed measurement were enrolled to this study.

Baltimore Longitudinal study on Aging (BLSA)

The Baltimore longitudinal study on Aging (BLSA) study is a population-based study aimed to evaluate contributors of healthy aging in the older population residing predominantly in the Baltimore-Washington DC area [4]. Starting in 1958, participants are examined every one to four years depending on their age. Currently there are approximately 1100 active participants enrolled in the study. Blood samples were collected for DNA extraction, and genome-wide genotyping was completed for 1231 subjects using Illumina 550K. This analysis focused on a subset of the participants (N=334) with European ancestry with data on walking speed (6 meter walk in normal pace). The BLSA has continuing approval from the Institutional Review Board (IRB) of Medstar Research Institute.

Cardiovascular Health Study (CHS)

The CHS is a population-based cohort study of risk factors for CHD and stroke in adults ≥65 years conducted across four field centers [5].The original predominantly Caucasian cohort of 5,201 persons was recruited in 1989-1990 from random samples of the Medicare eligibility lists; subsequently, an additional predominantly African-American cohort of 687 persons were enrolled for a total sample of 5,888. Only 3980 CHS participants who were free of CVD at baseline, consented to genetic testing, and had DNA available for genotyping were GWASed. Finally, to maintain race homogeneity we picked 3184 Caucasian with gait speed (4.6 meter walk normal pace) and genome wide assessments to participate in the current study.

Framingham Heart Study (FHS)

The FHS is a longitudinal community-based multi-generational study funded by the National Heart Lung and Blood Institute [6]. The Original cohort (Gen1) has undergone 32 biennial examinations since 1948; the Offspring cohort (Gen2) has participated in 9 exams from 1971 onwards, and the Omni group 1 cohort in 4 examinations from 1994 onwards. The Gen3 and Omni group 2 cohorts completed 2 examinations since 2002 and are currently starting the third examination cycle (April 2016). All participants undergo extensive research examinations and surviving Original cohort, Offspring and Gen 3 participants had genome-wide genotyping with the Affymetrix 500K Array Set and 50K Human Gene Focused Panel available at the start of this study [7]. At Offspring exam 8 (2005-2008) and Original cohort exam 26 (1999-2001), participants were asked to walk a 4 meter course at a normal pace while being timed with a stop watch by trained technicians. The usual pace walk was repeated and the faster of the two walks was used for analysis. Participants were excluded if under age 60. The final sample included 2384 participants (56.1% women), mean age 72.4 (SD 8.5) years (range 60 to 98) with gait speed and genomic

genotyping assessed. Informed consent was obtained at each attended exam and the Boston University Medical Center Institutional Review Board approved the protocol for all examinations.

Health, Aging, and Body Composition Study (HABC)

The Health Aging and Body Composition (HABC) Study is a NIA-sponsored cohort study of the factors that contribute to incident disability and the decline in function of healthier older persons, with a particular emphasis on changes in body composition in old age. Between March 1997 and July 1998, 3075 70-79 year old community-dwelling adults (41% African-American) were recruited to participate in the Health ABC Study; characteristics of the cohort have been described elsewhere [8]. Medicare beneficiary listings were used to recruit in metropolitan areas surrounding Pittsburgh, Pennsylvania, and Memphis, Tennessee. Eligibility criteria included having no difficulty walking one-quarter of a mile, climbing 10 steps, or performing activities of daily living (transferring, bathing, dressing, and eating); no history of active treatment for cancer in the prior 3 years; and no plans to move from the area within 3 years. Genotyping was successful for 2,802 unrelated individuals (1663 Caucasians and 1139 African Americans). To reduce race bias we include only Caucasians of which 1482 have their gait speed assessed in normal pace (6 meter walk) have enrolled to the study.

Health and Retirement Study (HRS)

The Health and Retirement Study (HRS) is a longitudinal survey of a representative sample of Americans over the age of 50 [9]. The current sample is over 26,000 persons in 17,000 households. Respondents are interviewed every two years about income and wealth, health and use of health services, work and retirement, and family connections. DNA was extracted from saliva collected during a face-to-face interview in the respondents' homes. These data represent respondents who provided DNA samples and signed consent forms in 2006 and 2008. Gait speed was measured only on respondents ≥ 65 years of age. Respondents were removed if they had gait velocities <0.05 or gait velocities > 5sd from the mean. A total of 5,073 subjects who have both a measure of gait speed (2.5 meter walk at a normal pace) and high quality imputed genomic genotypes were included in the analysis.

Invecchiare in Chianti (InCHIANTI)

The InCHIANTI study is a population-based epidemiological study aimed at evaluating the factors that influence mobility in the older population living in the Chianti region in Tuscany, Italy [10]. The details of the study have been previously reported. Briefly, 1616 residents were selected from the population registry of Greve in Chianti (a rural area: 11,709 residents with 19.3% of the population greater than 65 years of age), and Bagno a Ripoli (Antella village near Florence; 4,704 inhabitants, with 20.3% greater than 65 years of age). The participation rate was 90% (n=1453), and the subjects ranged between 21-102 years of age. Overnight fasted blood samples were for genomic DNA extraction. Illumina Infinium HumanHap 550K SNP arrays were used for genotyping. Data from 898 subjects were used for this analysis with genetic and walking speed (4 meter walk in normal pace) data. The study protocol was approved by the Italian National Institute of Research and Care of Aging Institutional Review and Medstar Research Institute (Baltimore, MD).

Lothian Birth Cohorts 1921 (LBC1921) and 1936 (LBC1936)

The Lothian Birth Cohorts include surviving participants from the Scottish Mental Surveys of 1932 or 1947 (SMS1932 and SMS1947), having been born, respectively in 1921 (LBC1921) and 1936 (LBC1936) [11-13]. The LBC1921 cohort consists of 550 relatively healthy individuals, 316 females and 234 males, assessed on cognitive and medical traits at about 79 years of age. When tested, the sample had a mean age of 79.1 years (SD = 0.6). The LBC1936 consists of 1091 relatively healthy individuals assessed on cognitive and medical traits at about 70 years of age. At baseline the sample of 548 men and 543 women had a mean age 69.6 years (SD = 0.8). They were all Caucasian and almost all lived independently in the Lothian region (Edinburgh city and surrounding area) of Scotland. Genotyping was performed at the Wellcome Trust Clinical Research Facility, Edinburgh. Among participants with genome-wide data and gait speed assessment (6 meter walk in normal pace), 510 (LBC1921) and 1001 (LBC1936) individuals were available for the present analysis.

Osteoporotic Fractures in Men Study (MrOS)

The Osteoporotic Fractures in Men Study (MrOS) is a multi-center prospective, longitudinal, observational study of risk factors for vertebral and all non-vertebral fractures in older men, and of the sequelae of fractures in men [14, 15]. MrOS study population consists of 5,994 community dwelling, ambulatory men aged 65 years or older from six communities in the United States (Birmingham, AL; Minneapolis, MN; Palo Alto, CA; Monongahela Valley near Pittsburgh, PA; Portland, OR; and San Diego, CA). Inclusion criteria were designed to provide a study cohort that is representative

of the broad population of older men. Genomic DNA from participants in the Osteoporotic Fractures in Men (MrOS) Study was extracted from whole blood samples collected at the baseline visit using the Flexigene protocol (Qiagen, Valencia, CA, USA) at the University of Pittsburgh. Among the 5994 MrOS participants enrolled at the baseline visit, 5130 samples with whole genome genotyping data that passed QC. Of which, only 4,643 with gait speed assessed in normal pace (6 meter walk) were enrolled to the study.

The Religious Orders Study and Rush Memory and Aging Project (ROSMAP)

Data came from 2 community based cohort studies of aging and dementia, the Religious Orders Study and Rush Memory and Aging Project (ROSMAP). Details about the study design have been described previously [16, 17]. Both studies were approved by the institutional review board of Rush University Medical Center. Participants were free of known dementia at enrollment and agreed to annual clinical evaluation and brain donation at the time of death. An informed consent and an Anatomic Gift Act form were obtained from each participant. The follow-up rate among the survivors exceeds 90%. The two studies are conducted by the same team of investigators and share a large common core of test batteries, which allows combined analysis of the data. Gait speed was derived by timing with a stop watch how long it took a participant to walk 8 feet (2.5m) at their usual pace [18]. DNA was extracted from whole blood, lymphocytes, or frozen postmortem brain tissue. Genotyping was performed at the Broad Institute's Center for Genotyping and the Translational Genomics Research Institute [19]. Among participants with genome-wide data and gait speed assessment 1,646 individuals were available for the present study.

Rotterdam Study (RSI, -II, -III)

The Rotterdam Study is a population-based study in Rotterdam that currently investigates 14,926 inhabitants from a suburb of the city aged 45 years or over. Participants were enrolled during three recruitment phases – in 1990 (cohort 1), 2000 (cohort 2), and 2006 (cohort 3) [20, 21]. Visits to the research center are planned every 3-4 years for various medical examinations. Genotyping was successfully performed on 11,496 participants. Gait assessment was introduced in the study protocol in 2009. The Rotterdam Study has been approved by the Medical Ethics Committee of the Erasmus MC and by the Ministry of Health, Welfare and Sport of the Netherlands , implementing the Wet Bevolkingsonderzoek: ERGO (Population Studies Act: Rotterdam Study). All participants provided written informed consent to participate in the study and to obtain information from their treating physicians. Gait assessment of 3651 subjects included 5.79-m long pressure-activated walkway (GAITRite Platinum; CIR systems, Sparta, NJ: 4.88-m active area; 120-Hz sampling rate) [22, 23]. Follow thorough exclusion the reminder 2911 subjects were genomic genotyped and imputed to the HapMap 2 reference panel.

Study of Osteoporotic Fractures (SOF)

The Study of Osteoporotic Fractures (SOF) is a prospective multicenter study of risk factors for vertebral and non-vertebral fractures [24]. The cohort is comprised of 9704 community dwelling women 65 years old or older recruited from populations-based listings in four U.S. areas: Baltimore, Maryland; Minneapolis, Minnesota; Portland, Oregon; and the Monongahela Valley, Pennsylvania. Women enrolled in the study were 99% Caucasian with African American women initially excluded from the study due to their low incidence of hip fractures. The SOF participants were followed up every four months by postcard or telephone to ascertain the occurrence of falls, fractures and changes in address. To date, follow-up rates have exceeded 95% for vital status and fractures, a review of pre-operative radiographs. The SOF study recruited only women. Among the 9704 SOF participants enrolled at the baseline visit, 3625 samples with whole genome genotyping data that passed QC. Of which, only 3,441 with gait speed assessed in normal pace (6 meter walk) were enrolled to the study.

Tasmanian Study of Cognition and Gait (TASCOG)

TASCOG is a study of cerebrovascular mechanisms underlying gait, balance and cognition in a population-based sample of Tasmanian people aged at least 60 years [25]. Individuals aged 60–86 years (n = 395) living in Southern Tasmania, Australia, were randomly selected from the electoral roll between 2006 and 2008 to participate in the study. Individuals were excluded if they lived in a nursing home, had a contraindication for magnetic resonance scanning (MRI) or were unable to walk without a gait aid. The response rate was 55%, and genotyping was performed at the Diamantina Institute, University of Queensland. The study was approved by the Human Health and Medical Research Ethics Committee, University of Tasmania. Genomic data and gait speed assessment (GAITRite) were available for 360 subjects that are part of this study.

Genetic Epidemiology Network of Arteriopathy (GENOA)

The Genetic Epidemiology Network of Arteriopathy (GENOA) study consists of hypertensive sibships

recruited for linkage and association studies in order to identify genes that influence blood pressure and its target organ damage [26]. In the initial phase of the GENOA study (Phase I: 1996-2001), all members of sibships containing ≥ 2 individuals with essential hypertension clinically diagnosed before age 60 were invited to participate, including both hypertensive and normotensive siblings. In the second phase of the GENOA study (Phase II: 2000-2004), 1239 European American participants were successfully re-recruited to measure potential target organ damage due to hypertension. From 2001-2006, Phase II GENOA participants that had a sibling willing and eligible to participate underwent a neurocognitive testing battery to assess several domains of cognitive and neurological functioning, including the assessment of gait speed (N=967). Participants were excluded from this analysis if they were less than 60 years of age or gait velocities >1.9m/s. The sample includes 471 European Americans (55.0% female) with imputed genotypes and a measure of gait speed on a 25 foot (7.6 meter) walking course.

Leiden Longevity Study (LLS)

The LLS has been designed to investigate biomarkers of healthy ageing and longevity [27] and has been described in detail previously [28]. It is a family-based study consisting of 1,671 offspring of 421 nonagenarian sibling pairs of Dutch descent, and their 744 partners. DNA from the LLS was extracted from white blood cells at baseline using conventional methods and genotyping was performed with Illumina Human660W-Quad and OmniExpress BeadChips (Illumina, San Diego, CA, USA). Imputation was performed with IMPUTE using the HapMap 2 reference panel [29]. Walking speed at usual pace was determined over 4 meters. Among participants with genome-wide data and gait speed assessment 235 individuals were available for the present study.

Osteoporotic Fractures in Men Study (MrOS) Sweden (Malmö[MrOSMalmo] and Gothenburg [MrOSGBG])

The Osteoporotic Fractures in Men (MrOS) study is a multicenter, prospective study including older men in Sweden, Hong Kong and the United States. The MrOS Sweden study (n=3014) [30] consists of three sub-cohorts from three different Swedish cities (n=1005 in Malmö, n=1010 in Gothenburg, and n=999 in Uppsala). Study subjects (men aged 69 to 81 years) were randomly identified using national population registers.
A total of 62% of the MrOS Sweden subjects who have both GAIT information and high quality imputed genomic genotypes participated in the study (n=922 in Malmö, n=960 in Gothenburg). To be eligible for the study, the subjects had to be able to walk without assistance, provide self-reported data, and sign an informed consent. The study was approved by the ethics committees at the Universities of Gothenburg, Lund, and Uppsala. Informed consent was obtained from all study participants. Genome-wide genotyping was performed in the MrOS Gothenburg and MrOS Malmö sub cohorts. Walking speed at usual pace was determined over 6 meters. Both duration of the walk and the number of steps were measured.

Expression quantitative trait loci (eQTL) analysis

A general overview of a subset of >50 eQTL studies has been published [31], with specific citations for >100 datasets included in the current query following here.
Blood cell related eQTL studies included fresh lymphocytes [32], fresh leukocytes [33], leukocyte samples in individuals with Celiac disease [34], whole blood samples [35-54], lymphoblastoid cell lines (LCL) derived from asthmatic children [55, 56], HapMap LCL from 3 populations [57], a separate study on HapMap CEU LCL [58], additional LCL population samples [59-65], neutrophils [66, 67], CD19+ B cells [68], primary PHA-stimulated T cells [62, 65], CD4+ T cells (20833654), peripheral blood monocytes [59, 68-72], long non-coding RNAs in monocytes [73] and CD14+ monocytes before and after stimulation with LPS or interferon-gamma [74], CD11+ dendritic cells before and after Mycobacterium tuberculosis infection [75] and a separate study of dendritic cells before or after stimulation with LPS, influenza or interferon-beta [76]. Micro-RNA QTLs [77, 78], DNase-I QTLs [79], histone acetylation QTLs [80], and ribosomal occupancy QTLs [81] were also queried for LCL. Splicing QTLs [82] and micro-RNA QTLs [83] were queried in whole blood.

Non-blood cell tissue eQTLs searched included omental and subcutaneous adipose [37, 48, 54, 63, 84], visceral fat [37] stomach [84], endometrial carcinomas [85], ER+ and ER- breast cancer tumor cells [86], liver [37, 84, 87-90], osteoblasts [91], intestine [92] and normal and cancerous colon [93, 94], skeletal muscle [37, 95], breast tissue (normal and cancer] [96, 97], lung [48, 98-101], skin [48, 59, 63, 102], primary fibroblasts [62, 65, 103], sputum [104], pancreatic islet cells [105], prostate [106], rectal mucosa [107], arterial wall [37] and heart tissue from left ventricles [48, 108] and left and right atria [109]. Micro-RNA QTLs were also queried for gluteal and abdominal adipose [110] and liver [111]. Methylation QTLs were queried in pancreatic islet cells [112]. Further mRNA and micro-RNA QTLs were queried from ER+ invasive breast cancer samples, colon-, kidney renal clear-, lung- and prostate-adenocarcinoma samples [113].

Brain eQTL studies included brain cortex [36, 72, 114-116], cerebellar cortex [117], cerebellum [115, 118-121], frontal cortex [117, 119, 121], gliomas [122], hippocampus [117, 119], inferior olivary nucleus (from medulla) [117], intralobular white matter [117], occipital cortex [117], parietal lobe [120], pons [121], pre-frontal cortex [118, 119, 123, 124], putamen (at the level of anterior commissure) [117], substantia nigra [117], temporal cortex [115, 117, 119, 121], thalamus [119] and visual cortex [118].

Additional eQTL data was integrated from online sources including ScanDB, the Broad Institute GTEx Portal, and the Pritchard Lab (eqtl.uchicago.edu). Cerebellum, parietal lobe and liver eQTL data was downloaded from ScanDB and cis-eQTLs were limited to those with P<1.0E-6 and trans-eQTLs with P<5.0E-8. Results for GTEx Analysis V4 for 13 tissues were downloaded from the GTEx Portal and then additionally filtered as described below www.gtexportal.org: thyroid, leg skin (sun exposed), tibial nerve, aortic artery, tibial artery, skeletal muscle, esophagus mucosa, esophagus muscularis, lung, heart (left ventricle), stomach, whole blood, and subcutaneous adipose [48]. Splicing QTL (sQTL) results generated with sQTLseeker with false discovery rate P≤0.05 were retained. For all gene-level eQTLs, if at least 1 SNP passed the tissue-specific empirical threshold in GTEx, the best SNP for that eQTL was always retained. All gene-level eQTL SNPs with P<1.67E-11 were also retained, reflecting a global threshold correction of P=0.05/(30,000 genes X 1,000,000 tests).

Analysis Plan

1) Analysis Plan:

a. Imputation: all cohorts have imputed to HapMap, using either BimBam or MACH.

b. Cohort-specific analyses

i. Multiple linear regression of imputed SNPs on gait speed (m/s)

ii. All analyses will be sex-combined

iii. SNPs will be coded as additive model as a count of the number of variant alleles present (1 degree of freedom).

iv. Covariate adjustment:

1. age (at time of exam)
2. gender
3. study site (for cohorts with multiple sites)
4. principal components that control for population stratification (in some cohorts)
5. height
6. Osteoarthritis*

c. Meta-analysis: Inverse variance weighted meta-analysis to be performed on summary statistics of imputed data. Meta-analysis of gait speed outcome will be performed using a fixed effects model of beta estimates and standard errors from each cohort.

i. Significance threshold: A threshold of p-value 5x10-8 will be used to determine genome-wide statistical significance.

*Cohorts with Osteoarthritis measurement will add a variable yes/no converted to 1/0 for any sort of osteoarthritis and will provide two analyses one with and one without this variable.

– Cohorts without Osteoarthritis measurement will stick to the original analytic plan.

– Cohorts with Osteoarthritis measurement will provide:

I. Analysis which includes everyone with or without osteoarthritis.

II. Analysis only for the one without this variable.

d. Cohorts with mixed ethnicity will be stratified by race. Meta analysis will test both possibilities with the additional race as a separate cohort and without.

2) Data format: The data delivery format for the meta-analysis will be according to the CHARGE protocol for file sharing.

a. ShareSpaces, a secure web-based file-sharing system implemented by the University of Washington's Catalyst computing group, will be used.

b. The following variables should be included when sharing imputed results for meta-analysis (Table 11).

SUPPLEMENTARY FIGURES

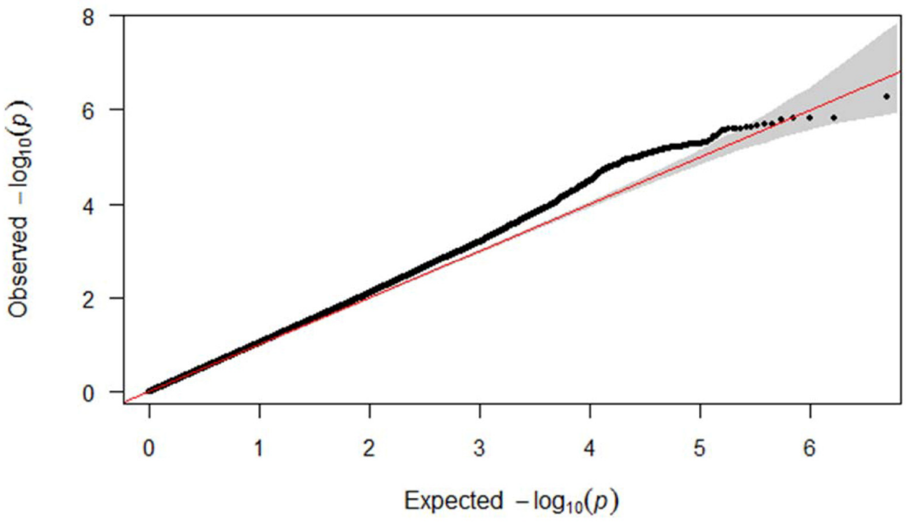

Supplementary Figure 1. Q-Q plot of expected (red line) vs. observed (black dot line) –log10 p-values for meta-analysis of genome-wide association studies of gait speed.

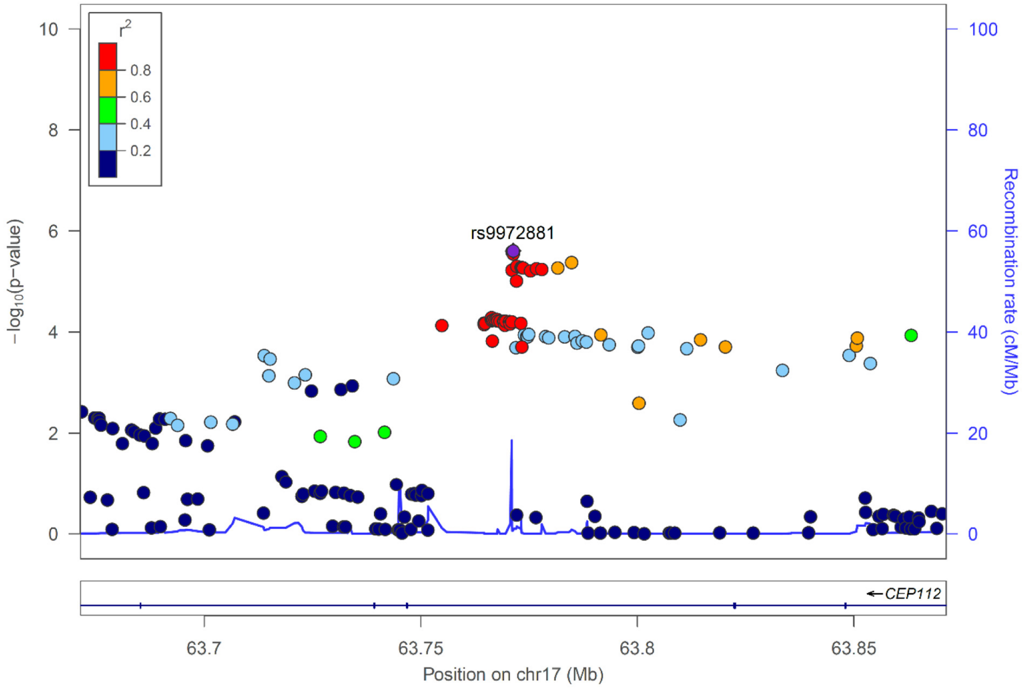

A

B

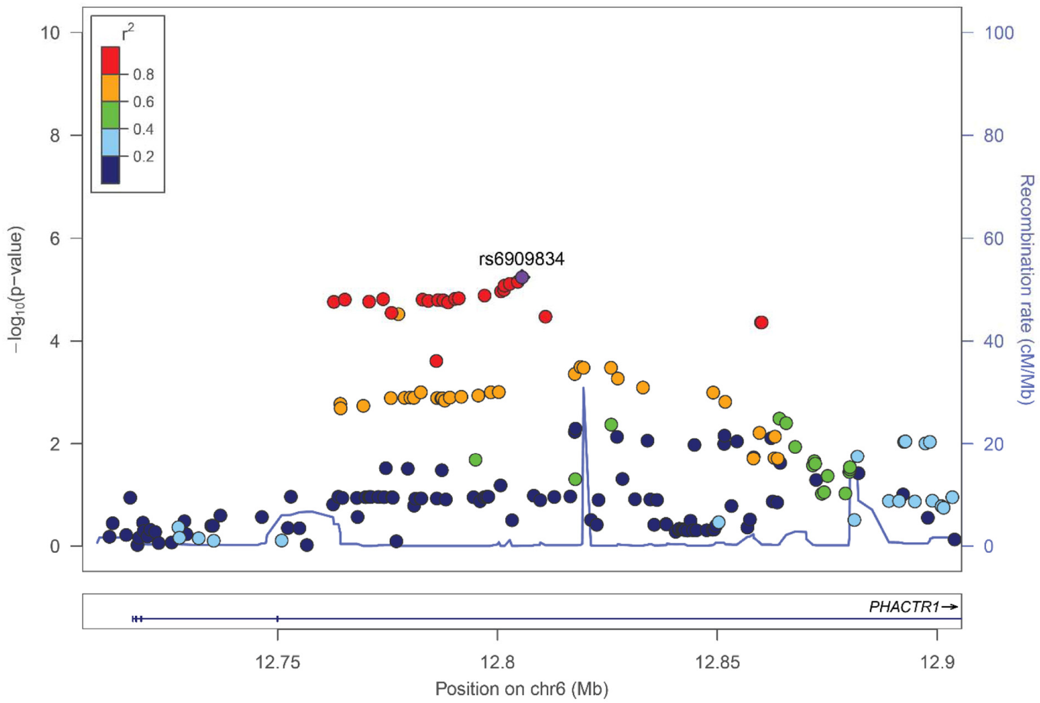

r2
0.8
0.6
0.4
0.2
rs6909834
−log10(p−value)
Recombination rate (cM/Mb)
PHACTR1→
12.75
12.8
12.85
12.9
Position on chr6 (Mb)

C

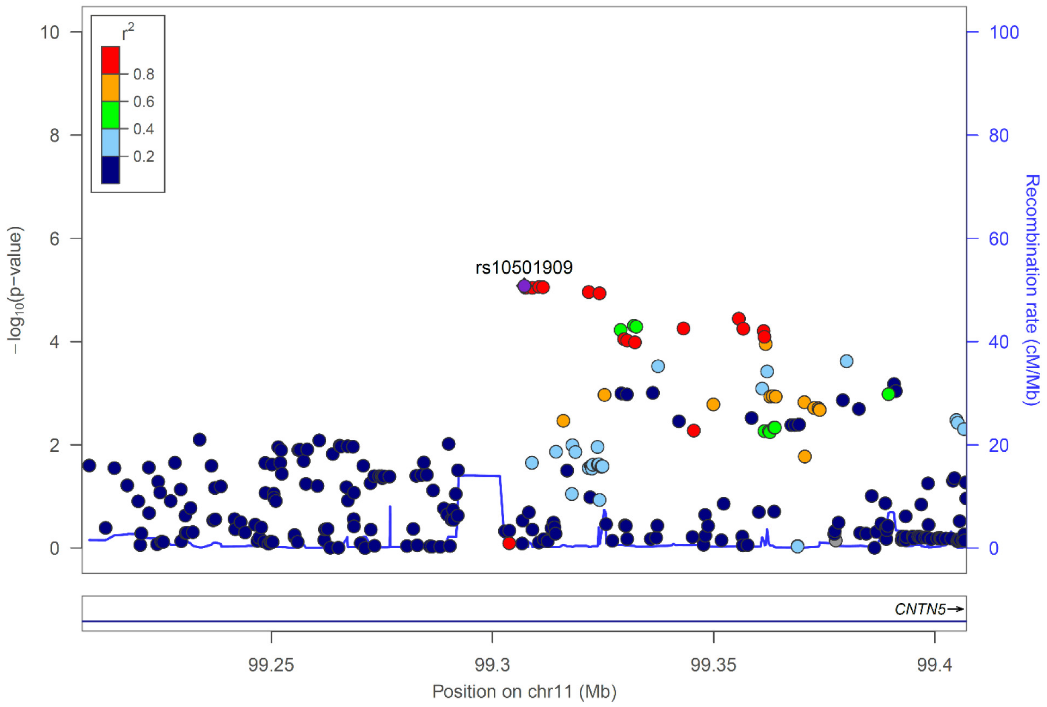

r2
0.8
0.6
0.4
0.2
rs10501909
−log10(p−value)
Recombination rate (cM/Mb)
CNTN5→
99.25
99.3
99.35
99.4
Position on chr11 (Mb)

D

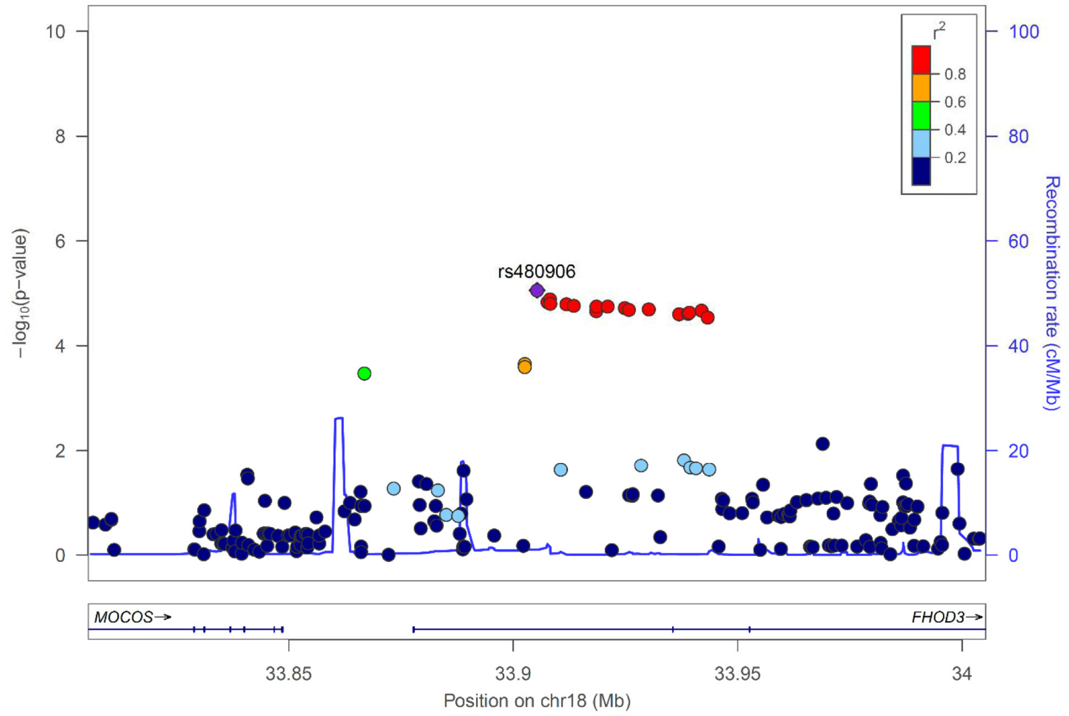
rs480906
r2
0.8
0.6
0.4
0.2
−log10(p−value)
Recombination rate (cM/Mb)
MOCOS
FHOD3
Position on chr18 (Mb)
33.85
33.9
33.95
34

E

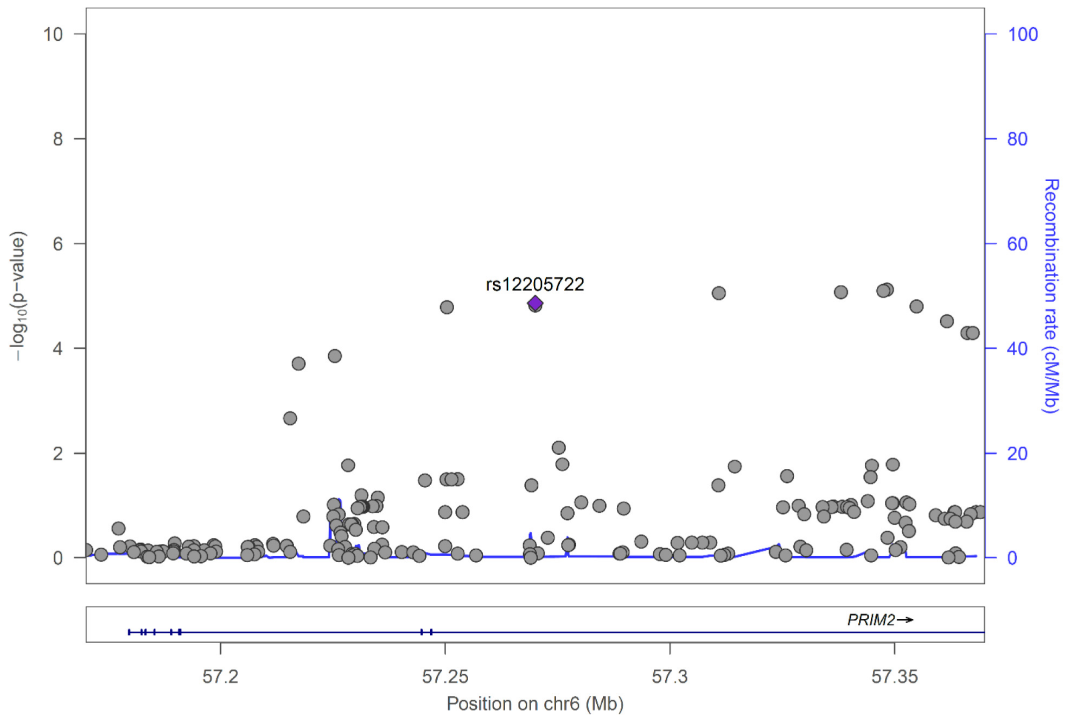
rs12205722
−log10(p−value)
Recombination rate (cM/Mb)
PRIM2
Position on chr6 (Mb)
57.2
57.25
57.3
57.35

F

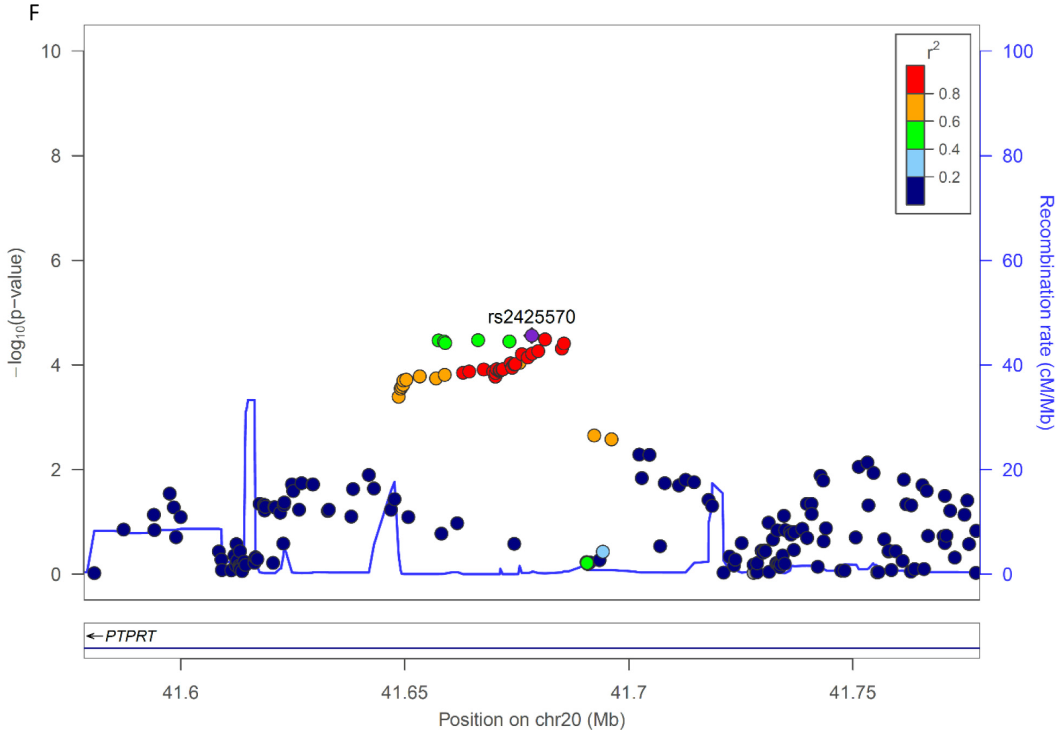

G

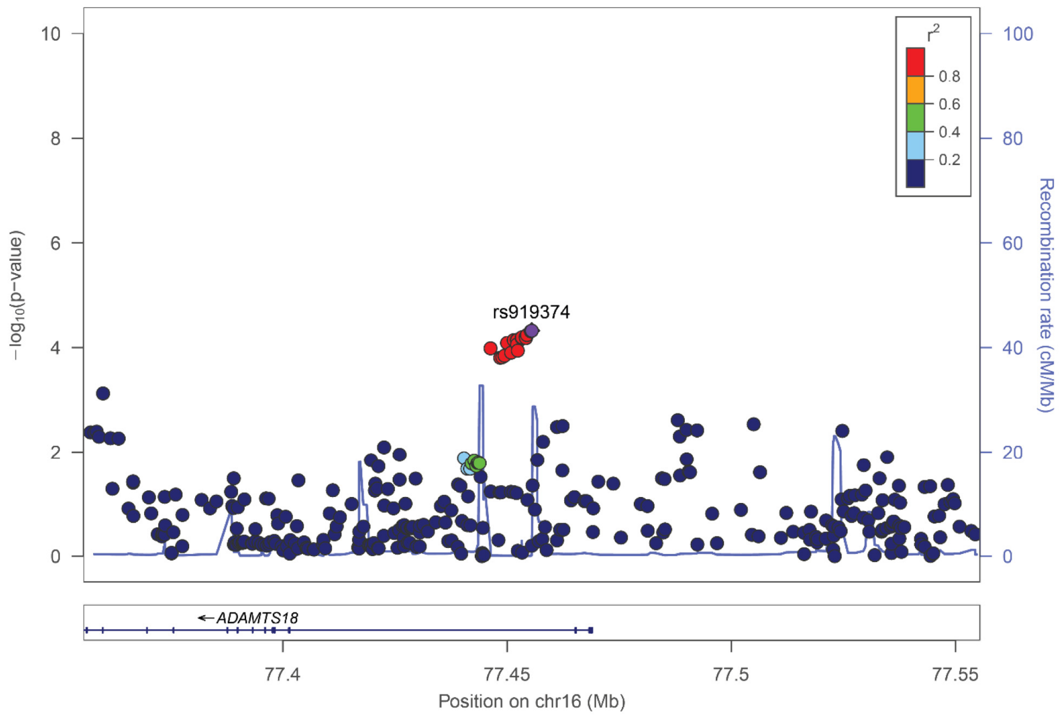

Supplementary Figure 2. LocusZoom plots for the genes (7 genes) with most suggestive variants (not listed in the top tens) associated with gait speed of the combined analysis (**A**) *CEP112*; (**B**) *PHACTR1*, (**C**) *CNTN5*, (**D**) *FHOD3*, (**E**) *PRIM2*, (**F**) *PTPRT*, (**G**) *ADAMTS18*). In each plot, the −log10 of *p* values are on the left *y*-axis; the SNP genomic position (HG19) on the *x*-axis; the estimated recombination rate from 1000 genomes Nov. 2014 EUR are on the right *y*-axis and plotted in blue. The most significant SNP is in purple diamond and plotted using the *p* value attained from the meta-analysis. SNPs are colored to reflect linkage disequilibrium (LD) with the most significant SNP in red (pairwise *r*2 from 1000 genomes Nov. 2014 EUR). Gene annotations are from the SeattleSeqAnnotation141.

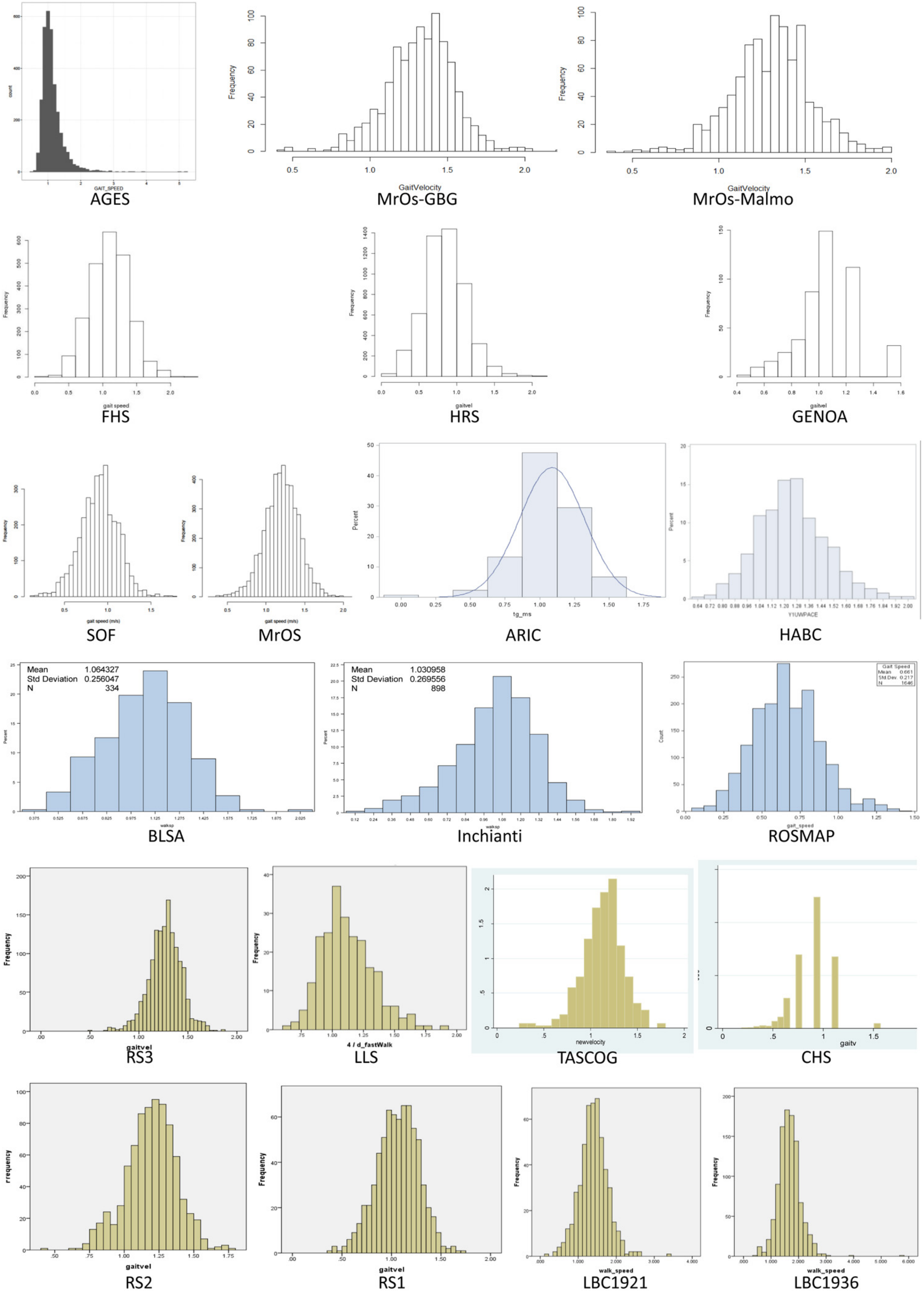

Supplementary Figure 3. Gait speed values distribution within cohorts.

SUPPLEMENTARY TABLES

Please browse links in Full Text version to see Supplementary Tables S1-S11.

SUPPLEMENTARY REFERENCES

1. Harris TB, Launer LJ, Eiriksdottir G, Kjartansson O, Jonsson PV, Sigurdsson G, Thorgeirsson G, Aspelund T, Garcia ME, Cotch MF, Hoffman HJ, Gudnason V. Age, Gene/Environment Susceptibility-Reykjavik Study: multidisciplinary applied phenomics. Am J Epidemiol. 2007; 165:1076–87. doi: 10.1093/aje/kwk115

2. The Atherosclerosis Risk in Communities (ARIC) Study: design and objectives. The ARIC investigators. Am J Epidemiol. 1989; 129:687–702. doi: 10.1093/oxfordjournals.aje.a115184

3. D. S. Knopman, T. H. Mosley, D. J. Catellier, L. H. Coker, M. R. I. S. Atherosclerosis Risk in Communities Study Brain, Fourteen-year longitudinal study of vascular risk factors, APOE genotype, and cognition: the ARIC MRI Study. Alzheimer's & dementia: the journal 5, 207-214 (2009).

4. Stone JL, Norris AH. Activities and attitudes of participants in the Baltimore longitudinal study. J Gerontol. 1966; 21:575–80. doi: 10.1093/geronj/21.4.575

5. Fried LP, Borhani NO, Enright P, Furberg CD, Gardin JM, Kronmal RA, Kuller LH, Manolio TA, Mittelmark MB, Newman A, O'Leary DH, Psaty B, Rautaharju P, et al. The Cardiovascular Health Study: design and rationale. Ann Epidemiol. 1991; 1:263–76. doi: 10.1016/1047-2797(91)90005-W

6. Splansky GL, Corey D, Yang Q, Atwood LD, Cupples LA, Benjamin EJ, D'Agostino RB Sr, Fox CS, Larson MG, Murabito JM, O'Donnell CJ, Vasan RS, Wolf PA, Levy D. The Third Generation Cohort of the National Heart, Lung, and Blood Institute's Framingham Heart Study: design, recruitment, and initial examination. Am J Epidemiol. 2007; 165:1328–35. doi: 10.1093/aje/kwm021

7. Benjamin IJ et al. American Heart Association Cardiovascular Genome-Phenome Study Circulation. 2014.

8. Harris TB, Visser M, Everhart J, Cauley J, Tylavsky F, Fuerst T, Zamboni M, Taaffe D, Resnick HE, Scherzinger A, Nevitt M. Waist circumference and sagittal diameter reflect total body fat better than visceral fat in older men and women. The Health, Aging and Body Composition Study. Ann N Y Acad Sci. 2000; 904:462–73. doi: 10.1111/j.1749-6632.2000.tb06501.x

9. Sonnega A, Faul JD, Ofstedal MB, Langa KM, Phillips JW, Weir DR. Cohort Profile: the Health and Retirement Study (HRS). Int J Epidemiol. 2014; 43:576–85. doi: 10.1093/ije/dyu067

10. Cesari M, Pahor M, Bartali B, Cherubini A, Penninx BW, Williams GR, Atkinson H, Martin A, Guralnik JM, Ferrucci L. Antioxidants and physical performance in elderly persons: the Invecchiare in Chianti (InCHIANTI) study. Am J Clin Nutr. 2004; 79:289–94.

11. Deary IJ, Whiteman MC, Starr JM, Whalley LJ, Fox HC. The impact of childhood intelligence on later life: following up the Scottish mental surveys of 1932 and 1947. J Pers Soc Psychol. 2004; 86:130–47. doi: 10.1037/0022-3514.86.1.130

12. Deary IJ, Gow AJ, Taylor MD, Corley J, Brett C, Wilson V, Campbell H, Whalley LJ, Visscher PM, Porteous DJ, Starr JM. The Lothian Birth Cohort 1936: a study to examine influences on cognitive ageing from age 11 to age 70 and beyond. BMC Geriatr. 2007; 7:28. doi: 10.1186/1471-2318-7-28

13. Deary IJ, Gow AJ, Pattie A, Starr JM. Cohort profile: the Lothian Birth Cohorts of 1921 and 1936. Int J Epidemiol. 2012; 41:1576–84. doi: 10.1093/ije/dyr197

14. Blank JB, Cawthon PM, Carrion-Petersen ML, Harper L, Johnson JP, Mitson E, Delay RR. Overview of recruitment for the osteoporotic fractures in men study (MrOS). Contemp Clin Trials. 2005; 26:557–68. doi: 10.1016/j.cct.2005.05.005

15. Orwoll E, Blank JB, Barrett-Connor E, Cauley J, Cummings S, Ensrud K, Lewis C, Cawthon PM, Marcus R, Marshall LM, McGowan J, Phipps K, Sherman S, et al. Design and baseline characteristics of the osteoporotic fractures in men (MrOS) study--a large observational study of the determinants of fracture in older men. Contemp Clin Trials. 2005; 26:569–85. doi: 10.1016/j.cct.2005.05.006

16. Bennett DA, Schneider JA, Arvanitakis Z, Wilson RS. Overview and findings from the religious orders study. Curr Alzheimer Res. 2012; 9:628–45. doi: 10.2174/156720512801322573

17. Bennett DA, Schneider JA, Buchman AS, Barnes LL, Boyle PA, Wilson RS. Overview and findings from the rush Memory and Aging Project. Curr Alzheimer Res. 2012; 9:646–63. doi: 10.2174/156720512801322663

18. Shah RC, Buchman AS, Boyle PA, Leurgans SE, Wilson RS, Andersson GB, Bennett DA. Musculoskeletal pain is associated with incident mobility disability in community-dwelling elders. J Gerontol A Biol Sci Med Sci. 2011; 66:82–88.

doi: 10.1093/gerona/glq187

19. Shulman JM, Chen K, Keenan BT, Chibnik LB, Fleisher A, Thiyyagura P, Roontiva A, McCabe C, Patsopoulos NA, Corneveaux JJ, Yu L, Huentelman MJ, Evans DA, et al. Genetic susceptibility for Alzheimer disease neuritic plaque pathology. JAMA Neurol. 2013; 70:1150–57. doi: 10.1001/jamaneurol.2013.2815

20. Hofman A, Breteler MM, van Duijn CM, Janssen HL, Krestin GP, Kuipers EJ, Stricker BH, Tiemeier H, Uitterlinden AG, Vingerling JR, Witteman JC. The Rotterdam Study: 2010 objectives and design update. Eur J Epidemiol. 2009; 24:553–72. doi: 10.1007/s10654-009-9386-z

21. Hofman A, Brusselle GG, Darwish Murad S, van Duijn CM, Franco OH, Goedegebure A, Ikram MA, Klaver CC, Nijsten TE, Peeters RP, Stricker BH, Tiemeier HW, Uitterlinden AG, Vernooij MW. The Rotterdam Study: 2016 objectives and design update. Eur J Epidemiol. 2015; 30:661–708. doi: 10.1007/s10654-015-0082-x

22. Verlinden VJ, van der Geest JN, Hoogendam YY, Hofman A, Breteler MM, Ikram MA. Gait patterns in a community-dwelling population aged 50 years and older. Gait Posture. 2013; 37:500–05. doi: 10.1016/j.gaitpost.2012.09.005

23. Verlinden VJ, van der Geest JN, Hofman A, Ikram MA. Cognition and gait show a distinct pattern of association in the general population. Alzheimers Dement. 2013.

24. Cummings SR, Nevitt MC, Browner WS, Stone K, Fox KM, Ensrud KE, Cauley J, Black D, Vogt TM, and Study of Osteoporotic Fractures Research Group. Risk factors for hip fracture in white women. N Engl J Med. 1995; 332:767–73. doi: 10.1056/NEJM199503233321202

25. Callisaya ML, Blizzard L, McGinley JL, Srikanth VK. Risk of falls in older people during fast-walking--the TASCOG study. Gait Posture. 2012; 36:510–15. doi: 10.1016/j.gaitpost.2012.05.003

26. Daniels PR, Kardia SL, Hanis CL, Brown CA, Hutchinson R, Boerwinkle E, Turner ST, and Genetic Epidemiology Network of Arteriopathy study. Familial aggregation of hypertension treatment and control in the Genetic Epidemiology Network of Arteriopathy (GENOA) study. Am J Med. 2004; 116:676–81. doi: 10.1016/j.amjmed.2003.12.032

27. Westendorp RG, van Heemst D, Rozing MP, Frölich M, Mooijaart SP, Blauw GJ, Beekman M, Heijmans BT, de Craen AJ, Slagboom PE, and Leiden Longevity Study Group. Nonagenarian siblings and their offspring display lower risk of mortality and morbidity than sporadic nonagenarians: The Leiden Longevity Study. J Am Geriatr Soc. 2009; 57:1634–37. doi: 10.1111/j.1532-5415.2009.02381.x

28. Schoenmaker M, de Craen AJ, de Meijer PH, Beekman M, Blauw GJ, Slagboom PE, Westendorp RG. Evidence of genetic enrichment for exceptional survival using a family approach: the Leiden Longevity Study. Eur J Hum Genet. 2006; 14:79–84.

29. Deelen J, Beekman M, Uh HW, Broer L, Ayers KL, Tan Q, Kamatani Y, Bennet AM, Tamm R, Trompet S, Guðbjartsson DF, Flachsbart F, Rose G, et al. Genome-wide association meta-analysis of human longevity identifies a novel locus conferring survival beyond 90 years of age. Hum Mol Genet. 2014; 23:4420–32. doi: 10.1093/hmg/ddu139

30. Ribom EL, Grundberg E, Mallmin H, Ohlsson C, Lorenzon M, Orwoll E, Holmberg AH, Mellström D, Ljunggren O, Karlsson MK. Estimation of physical performance and measurements of habitual physical activity may capture men with high risk to fall--data from the Mr Os Sweden cohort. Arch Gerontol Geriatr. 2009; 49:e72–76. doi: 10.1016/j.archger.2008.09.003

31. Zhang X, Gierman HJ, Levy D, Plump A, Dobrin R, Goring HH, Curran JE, Johnson MP, Blangero J, Kim SK, O'Donnell CJ, Emilsson V, Johnson AD. Synthesis of 53 tissue and cell line expression QTL datasets reveals master eQTLs. BMC Genomics. 2014; 15:532. doi: 10.1186/1471-2164-15-532

32. Göring HH, Curran JE, Johnson MP, Dyer TD, Charlesworth J, Cole SA, Jowett JB, Abraham LJ, Rainwater DL, Comuzzie AG, Mahaney MC, Almasy L, MacCluer JW, et al. Discovery of expression QTLs using large-scale transcriptional profiling in human lymphocytes. Nat Genet. 2007; 39:1208–16. doi: 10.1038/ng2119

33. Idaghdour Y, Czika W, Shianna KV, Lee SH, Visscher PM, Martin HC, Miclaus K, Jadallah SJ, Goldstein DB, Wolfinger RD, Gibson G. Geographical genomics of human leukocyte gene expression variation in southern Morocco. Nat Genet. 2010; 42:62–67. doi: 10.1038/ng.495

34. Heap GA, Trynka G, Jansen RC, Bruinenberg M, Swertz MA, Dinesen LC, Hunt KA, Wijmenga C, Vanheel DA, Franke L. Complex nature of SNP genotype effects on gene expression in primary human leucocytes. BMC Med Genomics. 2009; 2:1. doi: 10.1186/1755-8794-2-1

35. Chen W, Brehm JM, Lin J, Wang T, Forno E, Acosta-Pérez E, Boutaoui N, Canino G, Celedón JC. Expression quantitative trait loci (eQTL) mapping in Puerto Rican children. PLoS One. 2015; 10:e0122464. doi: 10.1371/journal.pone.0122464

36. Lock EF, Soldano KL, Garrett ME, Cope H, Markunas CA, Fuchs H, Grant G, Dunson DB, Gregory SG, Ashley-Koch AE. Joint eQTL assessment of whole blood and dura mater tissue from individuals with Chiari type I malformation. BMC Genomics. 2015; 16:11. doi: 10.1186/s12864-014-1211-8

37. Foroughi Asl H, Talukdar HA, Kindt AS, Jain RK, Ermel R, Ruusalepp A, Nguyen KD, Dobrin R, Reilly DF, Schunkert H, Samani NJ, Braenne I, Erdmann J, et al, and CARDIoGRAM Consortium. Expression quantitative trait Loci acting across multiple tissues are enriched in inherited risk for coronary artery disease. Circ Cardiovasc Genet. 2015; 8:305–15. doi: 10.1161/CIRCGENETICS.114.000640

38. Pierce BL, Tong L, Chen LS, Rahaman R, Argos M, Jasmine F, Roy S, Paul-Brutus R, Westra HJ, Franke L, Esko T, Zaman R, Islam T, et al. Mediation analysis demonstrates that trans-eQTLs are often explained by cis-mediation: a genome-wide analysis among 1,800 South Asians. PLoS Genet. 2014; 10:e1004818. doi: 10.1371/journal.pgen.1004818

39. Narahara M, Higasa K, Nakamura S, Tabara Y, Kawaguchi T, Ishii M, Matsubara K, Matsuda F, Yamada R. Large-scale East-Asian eQTL mapping reveals novel candidate genes for LD mapping and the genomic landscape of transcriptional effects of sequence variants. PLoS One. 2014; 9:e100924. doi: 10.1371/journal.pone.0100924

40. Schramm K, Marzi C, Schurmann C, Carstensen M, Reinmaa E, Biffar R, Eckstein G, Gieger C, Grabe HJ, Homuth G, Kastenmüller G, Mägi R, Metspalu A, et al. Mapping the genetic architecture of gene regulation in whole blood. PLoS One. 2014; 9:e93844. doi: 10.1371/journal.pone.0093844

41. Wright FA, Sullivan PF, Brooks AI, Zou F, Sun W, Xia K, Madar V, Jansen R, Chung W, Zhou YH, Abdellaoui A, Batista S, Butler C, et al. Heritability and genomics of gene expression in peripheral blood. Nat Genet. 2014; 46:430–37. doi: 10.1038/ng.2951

42. Quinlan J, Idaghdour Y, Goulet JP, Gbeha E, de Malliard T, Bruat V, Grenier JC, Gomez S, Sanni A, Rahimy MC, Awadalla P. Genomic architecture of sickle cell disease in West African children. Front Genet. 2014; 5:26. doi: 10.3389/fgene.2014.00026

43. Benton MC, Lea RA, Macartney-Coxson D, Carless MA, Göring HH, Bellis C, Hanna M, Eccles D, Chambers GK, Curran JE, Harper JL, Blangero J, Griffiths LR. Mapping eQTLs in the Norfolk Island genetic isolate identifies candidate genes for CVD risk traits. Am J Hum Genet. 2013; 93:1087–99. doi: 10.1016/j.ajhg.2013.11.004

44. Battle A, Mostafavi S, Zhu X, Potash JB, Weissman MM, McCormick C, Haudenschild CD, Beckman KB, Shi J, Mei R, Urban AE, Montgomery SB, Levinson DF, Koller D. Characterizing the genetic basis of transcriptome diversity through RNA-sequencing of 922 individuals. Genome Res. 2014; 24:14–24. doi: 10.1101/gr.155192.113

45. Westra HJ, Peters MJ, Esko T, Yaghootkar H, Schurmann C, Kettunen J, Christiansen MW, Fairfax BP, Schramm K, Powell JE, Zhernakova A, Zhernakova DV, Veldink JH, et al. Systematic identification of trans eQTLs as putative drivers of known disease associations. Nat Genet. 2013; 45:1238–43. doi: 10.1038/ng.2756

46. Landmark-Høyvik H, Dumeaux V, Nebdal D, Lund E, Tost J, Kamatani Y, Renault V, Børresen-Dale AL, Kristensen V, Edvardsen H. Genome-wide association study in breast cancer survivors reveals SNPs associated with gene expression of genes belonging to MHC class I and II. Genomics. 2013; 102:278–87. doi: 10.1016/j.ygeno.2013.07.006

47. Zhernakova DV, de Klerk E, Westra HJ, Mastrokolias A, Amini S, Ariyurek Y, Jansen R, Penninx BW, Hottenga JJ, Willemsen G, de Geus EJ, Boomsma DI, Veldink JH, et al. DeepSAGE reveals genetic variants associated with alternative polyadenylation and expression of coding and non-coding transcripts. PLoS Genet. 2013; 9:e1003594. doi: 10.1371/journal.pgen.1003594

48. Consortium GT, and GTEx Consortium. (GTEx) project. Nat Genet. 2013; 45:580–85. doi: 10.1038/ng.2653

49. Sasayama D, Hori H, Nakamura S, Miyata R, Teraishi T, Hattori K, Ota M, Yamamoto N, Higuchi T, Amano N, Kunugi H. Identification of single nucleotide polymorphisms regulating peripheral blood mRNA expression with genome-wide significance: an eQTL study in the Japanese population. PLoS One. 2013; 8:e54967. doi: 10.1371/journal.pone.0054967

50. van Eijk KR, de Jong S, Boks MP, Langeveld T, Colas F, Veldink JH, de Kovel CG, Janson E, Strengman E, Langfelder P, Kahn RS, van den Berg LH, Horvath S, Ophoff RA. Genetic analysis of DNA methylation and gene expression levels in whole blood of healthy human subjects. BMC Genomics. 2012; 13:636. doi: 10.1186/1471-2164-13-636

51. Mehta D, Heim K, Herder C, Carstensen M, Eckstein G, Schurmann C, Homuth G, Nauck M, Völker U, Roden M, Illig T, Gieger C, Meitinger T, Prokisch H. Impact of common regulatory single-nucleotide variants on gene expression profiles in whole blood. Eur J Hum Genet. 2013; 21:48–54. doi: 10.1038/ejhg.2012.106

52. Powell JE, Henders AK, McRae AF, Caracella A, Smith S, Wright MJ, Whitfield JB, Dermitzakis ET, Martin NG, Visscher PM, Montgomery GW. The Brisbane Systems Genetics Study: genetical genomics meets complex trait genetics. PLoS One. 2012; 7:e35430. doi: 10.1371/journal.pone.0035430

53. Fehrmann RS, Jansen RC, Veldink JH, Westra HJ, Arends D, Bonder MJ, Fu J, Deelen P, Groen HJ, Smolonska A, Weersma RK, Hofstra RM, Buurman WA, et al. Trans-eQTLs reveal that independent genetic variants associated with a complex phenotype converge on intermediate genes, with a major role for the HLA. PLoS Genet. 2011; 7:e1002197. doi: 10.1371/journal.pgen.1002197

54. Emilsson V, Thorleifsson G, Zhang B, Leonardson AS, Zink F, Zhu J, Carlson S, Helgason A, Walters GB, Gunnarsdottir S, Mouy M, Steinthorsdottir V, Eiriksdottir GH, et al. Genetics of gene expression and its effect on disease. Nature. 2008; 452:423–28. doi: 10.1038/nature06758

55. Liang L, Morar N, Dixon AL, Lathrop GM, Abecasis GR, Moffatt MF, Cookson WO. A cross-platform analysis of 14,177 expression quantitative trait loci derived from lymphoblastoid cell lines. Genome Res. 2013; 23:716–26. doi: 10.1101/gr.142521.112

56. Dixon AL, Liang L, Moffatt MF, Chen W, Heath S, Wong KC, Taylor J, Burnett E, Gut I, Farrall M, Lathrop GM, Abecasis GR, Cookson WO. A genome-wide association study of global gene expression. Nat Genet. 2007; 39:1202–07. doi: 10.1038/ng2109

57. Stranger BE, Nica AC, Forrest MS, Dimas A, Bird CP, Beazley C, Ingle CE, Dunning M, Flicek P, Koller D, Montgomery S, Tavaré S, Deloukas P, Dermitzakis ET. Population genomics of human gene expression. Nat Genet. 2007; 39:1217–24. doi: 10.1038/ng2142

58. Kwan T, Benovoy D, Dias C, Gurd S, Provencher C, Beaulieu P, Hudson TJ, Sladek R, Majewski J. Genome-wide analysis of transcript isoform variation in humans. Nat Genet. 2008; 40:225–31. doi: 10.1038/ng.2007.57

59. Huang J, Chen J, Esparza J, Ding J, Elder JT, Abecasis GR, Lee YA, Mark Lathrop G, Moffatt MF, Cookson WO, Liang L. eQTL mapping identifies insertion- and deletion-specific eQTLs in multiple tissues. Nat Commun. 2015; 6:6821. doi: 10.1038/ncomms7821

60. Bryois J, Buil A, Evans DM, Kemp JP, Montgomery SB, Conrad DF, Ho KM, Ring S, Hurles M, Deloukas P, Davey Smith G, Dermitzakis ET. Cis and trans effects of human genomic variants on gene expression. PLoS Genet. 2014; 10:e1004461. doi: 10.1371/journal.pgen.1004461

61. Mangravite LM, Engelhardt BE, Medina MW, Smith JD, Brown CD, Chasman DI, Mecham BH, Howie B, Shim H, Naidoo D, Feng Q, Rieder MJ, Chen YD, et al. A statin-dependent QTL for GATM expression is associated with statin-induced myopathy. Nature. 2013; 502:377–80. doi: 10.1038/nature12508

62. Gutierrez-Arcelus M, Lappalainen T, Montgomery SB, Buil A, Ongen H, Yurovsky A, Bryois J, Giger T, Romano L, Planchon A, Falconnet E, Bielser D, Gagnebin M, et al. Passive and active DNA methylation and the interplay with genetic variation in gene regulation. eLife. 2013; 2:e00523.

63. Grundberg E, Small KS, Hedman ÅK, Nica AC, Buil A, Keildson S, Bell JT, Yang TP, Meduri E, Barrett A, Nisbett J, Sekowska M, Wilk A, et al, and Multiple Tissue Human Expression Resource (MuTHER) Consortium. Mapping cis- and trans-regulatory effects across multiple tissues in twins. Nat Genet. 2012; 44:1084–89. doi: 10.1038/ng.2394

64. Cusanovich DA, Billstrand C, Zhou X, Chavarria C, De Leon S, Michelini K, Pai AA, Ober C, Gilad Y. The combination of a genome-wide association study of lymphocyte count and analysis of gene expression data reveals novel asthma candidate genes. Hum Mol Genet. 2012; 21:2111–23. doi: 10.1093/hmg/dds021

65. Dimas AS, Deutsch S, Stranger BE, Montgomery SB, Borel C, Attar-Cohen H, Ingle C, Beazley C, Gutierrez Arcelus M, Sekowska M, Gagnebin M, Nisbett J, Deloukas P, et al. Common regulatory variation impacts gene expression in a cell type-dependent manner. Science. 2009; 325:1246–50. doi: 10.1126/science.1174148

66. Andiappan AK, Melchiotti R, Poh TY, Nah M, Puan KJ, Vigano E, Haase D, Yusof N, San Luis B, Lum J, Kumar D, Foo S, Zhuang L, et al. Genome-wide analysis of the genetic regulation of gene expression in human neutrophils. Nat Commun. 2015; 6:7971. doi: 10.1038/ncomms8971

67. Naranbhai V, Fairfax BP, Makino S, Humburg P, Wong D, Ng E, Hill AV, Knight JC. Genomic modulators of gene expression in human neutrophils. Nat Commun. 2015; 6:7545. doi: 10.1038/ncomms8545

68. Fairfax BP, Makino S, Radhakrishnan J, Plant K, Leslie S, Dilthey A, Ellis P, Langford C, Vannberg FO, Knight JC. Genetics of gene expression in primary immune cells identifies cell type-specific master regulators and roles of HLA alleles. Nat Genet. 2012; 44:502–10. doi: 10.1038/ng.2205

69. Kirsten H, Al-Hasani H, Holdt L, Gross A, Beutner F, Krohn K, Horn K, Ahnert P, Burkhardt R, Reiche K, Hackermüller J, Löffler M, Teupser D, et al.

Dissecting the genetics of the human transcriptome identifies novel trait-related trans-eQTLs and corroborates the regulatory relevance of non-protein coding loci. Hum Mol Genet. 2015; 24:4746–63. doi: 10.1093/hmg/ddv194

70. Almlöf JC, Lundmark P, Lundmark A, Ge B, Maouche S, Göring HH, Liljedahl U, Enström C, Brocheton J, Proust C, Godefroy T, Sambrook JG, Jolley J, et al. Powerful identification of cis-regulatory SNPs in human primary monocytes using allele-specific gene expression. PLoS One. 2012; 7:e52260. doi: 10.1371/journal.pone.0052260

71. Zeller T, Wild P, Szymczak S, Rotival M, Schillert A, Castagne R, Maouche S, Germain M, Lackner K, Rossmann H, Eleftheriadis M, Sinning CR, Schnabel RB, et al. Genetics and beyond--the transcriptome of human monocytes and disease susceptibility. PLoS One. 2010; 5:e10693. doi: 10.1371/journal.pone.0010693

72. Heinzen EL, Ge D, Cronin KD, Maia JM, Shianna KV, Gabriel WN, Welsh-Bohmer KA, Hulette CM, Denny TN, Goldstein DB. Tissue-specific genetic control of splicing: implications for the study of complex traits. PLoS Biol. 2008; 6:e1. doi: 10.1371/journal.pbio.1000001

73. Almlöf JC, Lundmark P, Lundmark A, Ge B, Pastinen T, Goodall AH, Cambien F, Deloukas P, Ouwehand WH, Syvänen AC, and Cardiogenics Consortium. Single nucleotide polymorphisms with cis-regulatory effects on long non-coding transcripts in human primary monocytes. PLoS One. 2014; 9:e102612. doi: 10.1371/journal.pone.0102612

74. Fairfax BP, Humburg P, Makino S, Naranbhai V, Wong D, Lau E, Jostins L, Plant K, Andrews R, McGee C, Knight JC. Innate immune activity conditions the effect of regulatory variants upon monocyte gene expression. Science. 2014; 343:1246949. doi: 10.1126/science.1246949

75. Barreiro LB, Tailleux L, Pai AA, Gicquel B, Marioni JC, Gilad Y. Deciphering the genetic architecture of variation in the immune response to Mycobacterium tuberculosis infection. Proc Natl Acad Sci USA. 2012; 109:1204–09. doi: 10.1073/pnas.1115761109

76. Lee MN, Ye C, Villani AC, Raj T, Li W, Eisenhaure TM, Imboywa SH, Chipendo PI, Ran FA, Slowikowski K, Ward LD, Raddassi K, McCabe C, et al. Common genetic variants modulate pathogen-sensing responses in human dendritic cells. Science. 2014; 343:1246980. doi: 10.1126/science.1246980

77. Fischer D, Wahlfors T, Mattila H, Oja H, Tammela TL, Schleutker J. MiRNA Profiles in Lymphoblastoid Cell Lines of Finnish Prostate Cancer Families. PLoS One. 2015; 10:e0127427. doi: 10.1371/journal.pone.0127427

78. Huang RS, Gamazon ER, Ziliak D, Wen Y, Im HK, Zhang W, Wing C, Duan S, Bleibel WK, Cox NJ, Dolan ME. Population differences in microRNA expression and biological implications. RNA Biol. 2011; 8:692–701. doi: 10.4161/rna.8.4.16029

79. Degner JF, Pai AA, Pique-Regi R, Veyrieras JB, Gaffney DJ, Pickrell JK, De Leon S, Michelini K, Lewellen N, Crawford GE, Stephens M, Gilad Y, Pritchard JK. DNase I sensitivity QTLs are a major determinant of human expression variation. Nature. 2012; 482:390–94. doi: 10.1038/nature10808

80. del Rosario RC, Poschmann J, Rouam SL, Png E, Khor CC, Hibberd ML, Prabhakar S. Sensitive detection of chromatin-altering polymorphisms reveals autoimmune disease mechanisms. Nat Methods. 2015; 12:458–64. doi: 10.1038/nmeth.3326

81. Battle A, Khan Z, Wang SH, Mitrano A, Ford MJ, Pritchard JK, Gilad Y. Genomic variation. Impact of regulatory variation from RNA to protein. Science. 2015; 347:664–67. doi: 10.1126/science.1260793

82. Zhang X, Joehanes R, Chen BH, Huan T, Ying S, Munson PJ, Johnson AD, Levy D, O'Donnell CJ. Identification of common genetic variants controlling transcript isoform variation in human whole blood. Nat Genet. 2015; 47:345–52. doi: 10.1038/ng.3220

83. Huan T, Rong J, Liu C, Zhang X, Tanriverdi K, Joehanes R, Chen BH, Murabito JM, Yao C, Courchesne P, Munson PJ, O'Donnell CJ, Cox N, et al. Genome-wide identification of microRNA expression quantitative trait loci. Nat Commun. 2015; 6:6601. doi: 10.1038/ncomms7601

84. Greenawalt DM, Dobrin R, Chudin E, Hatoum IJ, Suver C, Beaulaurier J, Zhang B, Castro V, Zhu J, Sieberts SK, Wang S, Molony C, Heymsfield SB, et al. A survey of the genetics of stomach, liver, and adipose gene expression from a morbidly obese cohort. Genome Res. 2011; 21:1008–16. doi: 10.1101/gr.112821.110

85. Kompass KS, Witte JS. Co-regulatory expression quantitative trait loci mapping: method and application to endometrial cancer. BMC Med Genomics. 2011; 4:6. doi: 10.1186/1755-8794-4-6

86. Li Q, Seo JH, Stranger B, McKenna A, Pe'er I, Laframboise T, Brown M, Tyekucheva S, Freedman ML. Integrative eQTL-based analyses reveal the biology of breast cancer risk loci. Cell. 2013; 152:633–41. doi: 10.1016/j.cell.2012.12.034

87. Wang X, Tang H, Teng M, Li Z, Li J, Fan J, Zhong L, Sun X, Xu J, Chen G, Chen D, Wang Z, Xing T, et al. Mapping of hepatic expression quantitative trait loci (eQTLs) in a Han Chinese population. J Med Genet. 2014; 51:319–26. doi: 10.1136/jmedgenet-2013-102045

88. Schröder A, Klein K, Winter S, Schwab M, Bonin M, Zell A, Zanger UM. Genomics of ADME gene expression: mapping expression quantitative trait loci relevant for absorption, distribution, metabolism and excretion of drugs in human liver. Pharmacogenomics J. 2013; 13:12–20. doi: 10.1038/tpj.2011.44

89. Innocenti F, Cooper GM, Stanaway IB, Gamazon ER, Smith JD, Mirkov S, Ramirez J, Liu W, Lin YS, Moloney C, Aldred SF, Trinklein ND, Schuetz E, et al. Identification, replication, and functional fine-mapping of expression quantitative trait loci in primary human liver tissue. PLoS Genet. 2011; 7:e1002078. doi: 10.1371/journal.pgen.1002078

90. Schadt EE, Molony C, Chudin E, Hao K, Yang X, Lum PY, Kasarskis A, Zhang B, Wang S, Suver C, Zhu J, Millstein J, Sieberts S, et al. Mapping the genetic architecture of gene expression in human liver. PLoS Biol. 2008; 6:e107. doi: 10.1371/journal.pbio.0060107

91. Grundberg E, Kwan T, Ge B, Lam KC, Koka V, Kindmark A, Mallmin H, Dias J, Verlaan DJ, Ouimet M, Sinnett D, Rivadeneira F, Estrada K, et al. Population genomics in a disease targeted primary cell model. Genome Res. 2009; 19:1942–52. doi: 10.1101/gr.095224.109

92. Kabakchiev B, Silverberg MS. Expression quantitative trait loci analysis identifies associations between genotype and gene expression in human intestine. Gastroenterology. 2013; 144:1488–96, 1496.e1–3. doi: 10.1053/j.gastro.2013.03.001

93. Hulur I, Gamazon ER, Skol AD, Xicola RM, Llor X, Onel K, Ellis NA, Kupfer SS. Enrichment of inflammatory bowel disease and colorectal cancer risk variants in colon expression quantitative trait loci. BMC Genomics. 2015; 16:138. doi: 10.1186/s12864-015-1292-z

94. Ongen H, Andersen CL, Bramsen JB, Oster B, Rasmussen MH, Ferreira PG, Sandoval J, Vidal E, Whiffin N, Planchon A, Padioleau I, Bielser D, Romano L, et al. Putative cis-regulatory drivers in colorectal cancer. Nature. 2014; 512:87–90.

95. Keildson S, Fadista J, Ladenvall C, Hedman ÅK, Elgzyri T, Small KS, Grundberg E, Nica AC, Glass D, Richards JB, Barrett A, Nisbet J, Zheng HF, et al, and MAGIC Consortium, and DIAGRAM Consortium, and MuTHER Consortium. Expression of phosphofructokinase in skeletal muscle is influenced by genetic variation and associated with insulin sensitivity. Diabetes. 2014; 63:1154–65. doi: 10.2337/db13-1301

96. Quigley DA, Fiorito E, Nord S, Van Loo P, Alnæs GG, Fleischer T, Tost J, Moen Vollan HK, Tramm T, Overgaard J, Bukholm IR, Hurtado A, Balmain A, et al. The 5p12 breast cancer susceptibility locus affects MRPS30 expression in estrogen-receptor positive tumors. Mol Oncol. 2014; 8:273–84. doi: 10.1016/j.molonc.2013.11.008

97. Curtis C, Shah SP, Chin SF, Turashvili G, Rueda OM, Dunning MJ, Speed D, Lynch AG, Samarajiwa S, Yuan Y, Gräf S, Ha G, Haffari G, et al, and METABRIC Group. The genomic and transcriptomic architecture of 2,000 breast tumours reveals novel subgroups. Nature. 2012; 486:346–52.

98. Luo W, et al. Airway Epithelial Expression Quantitative Trait Loci Reveal Genes Underlying Asthma and Other Airway Diseases. Am J Respir Cell Mol Biol. 2015.

99. Gao C, Tignor NL, Salit J, Strulovici-Barel Y, Hackett NR, Crystal RG, Mezey JG. HEFT: eQTL analysis of many thousands of expressed genes while simultaneously controlling for hidden factors. Bioinformatics. 2014; 30:369–76. doi: 10.1093/bioinformatics/btt690

100. Lamontagne M, Couture C, Postma DS, Timens W, Sin DD, Paré PD, Hogg JC, Nickle D, Laviolette M, Bossé Y. Refining susceptibility loci of chronic obstructive pulmonary disease with lung eqtls. PLoS One. 2013; 8:e70220. doi: 10.1371/journal.pone.0070220

101. Hao K, Bossé Y, Nickle DC, Paré PD, Postma DS, Laviolette M, Sandford A, Hackett TL, Daley D, Hogg JC, Elliott WM, Couture C, Lamontagne M, et al. Lung eQTLs to help reveal the molecular underpinnings of asthma. PLoS Genet. 2012; 8:e1003029. doi: 10.1371/journal.pgen.1003029

102. Ding J, Gudjonsson JE, Liang L, Stuart PE, Li Y, Chen W, Weichenthal M, Ellinghaus E, Franke A, Cookson W, Nair RP, Elder JT, Abecasis GR. Gene expression in skin and lymphoblastoid cells: refined statistical method reveals extensive overlap in cis-eQTL signals. Am J Hum Genet. 2010; 87:779–89. doi: 10.1016/j.ajhg.2010.10.024

103. Wagner JR, Busche S, Ge B, Kwan T, Pastinen T, Blanchette M. The relationship between DNA methylation, genetic and expression inter-individual variation in untransformed human fibroblasts. Genome Biol. 2014; 15:R37. doi: 10.1186/gb-2014-15-2-r37

104. Qiu W, Cho MH, Riley JH, Anderson WH, Singh D, Bakke P, Gulsvik A, Litonjua AA, Lomas DA, Crapo JD, Beaty TH, Celli BR, Rennard S, et al, and ECLIPSE Investigators. Genetics of sputum gene expression in chronic obstructive pulmonary disease. PLoS One. 2011; 6:e24395. doi: 10.1371/journal.pone.0024395

105. Fadista J, Vikman P, Laakso EO, Mollet IG, Esguerra JL, Taneera J, Storm P, Osmark P, Ladenvall C, Prasad RB, Hansson KB, Finotello F, Uvebrant K, et al. Global genomic and transcriptomic analysis of human pancreatic islets reveals novel genes influencing glucose metabolism. Proc Natl Acad Sci USA. 2014; 111:13924–29. doi: 10.1073/pnas.1402665111

106. Larson NB, McDonnell S, French AJ, Fogarty Z, Cheville J, Middha S, Riska S, Baheti S, Nair AA, Wang L, Schaid DJ, Thibodeau SN. Comprehensively evaluating cis-regulatory variation in the human prostate transcriptome by using gene-level allele-specific expression. Am J Hum Genet. 2015; 96:869–82. doi: 10.1016/j.ajhg.2015.04.015

107. Singh T, Levine AP, Smith PJ, Smith AM, Segal AW, Barrett JC. Characterization of expression quantitative trait loci in the human colon. Inflamm Bowel Dis. 2015; 21:251–56. doi: 10.1097/MIB.0000000000000265

108. Koopmann TT, Adriaens ME, Moerland PD, Marsman RF, Westerveld ML, Lal S, Zhang T, Simmons CQ, Baczko I, dos Remedios C, Bishopric NH, Varro A, George AL Jr, et al. Genome-wide identification of expression quantitative trait loci (eQTLs) in human heart. PLoS One. 2014; 9:e97380. doi: 10.1371/journal.pone.0097380

109. Lin H, Dolmatova EV, Morley MP, Lunetta KL, McManus DD, Magnani JW, Margulies KB, Hakonarson H, del Monte F, Benjamin EJ, Cappola TP, Ellinor PT. Gene expression and genetic variation in human atria. Heart Rhythm. 2014; 11:266–71. doi: 10.1016/j.hrthm.2013.10.051

110. Rantalainen M, Herrera BM, Nicholson G, Bowden R, Wills QF, Min JL, Neville MJ, Barrett A, Allen M, Rayner NW, Fleckner J, McCarthy MI, Zondervan KT, et al. MicroRNA expression in abdominal and gluteal adipose tissue is associated with mRNA expression levels and partly genetically driven. PLoS One. 2011; 6:e27338. doi: 10.1371/journal.pone.0027338

111. Gamazon ER, Innocenti F, Wei R, Wang L, Zhang M, Mirkov S, Ramírez J, Huang RS, Cox NJ, Ratain MJ, Liu W. A genome-wide integrative study of microRNAs in human liver. BMC Genomics. 2013; 14:395. doi: 10.1186/1471-2164-14-395

112. Olsson AH, Volkov P, Bacos K, Dayeh T, Hall E, Nilsson EA, Ladenvall C, Rönn T, Ling C. Genome-wide associations between genetic and epigenetic variation influence mRNA expression and insulin secretion in human pancreatic islets. PLoS Genet. 2014; 10:e1004735. doi: 10.1371/journal.pgen.1004735

113. Li Q, Stram A, Chen C, Kar S, Gayther S, Pharoah P, Haiman C, Stranger B, Kraft P, Freedman ML. Expression QTL-based analyses reveal candidate causal genes and loci across five tumor types. Hum Mol Genet. 2014; 23:5294–302. doi: 10.1093/hmg/ddu228

114. Kim Y, Xia K, Tao R, Giusti-Rodriguez P, Vladimirov V, van den Oord E, Sullivan PF. A meta-analysis of gene expression quantitative trait loci in brain. Transl Psychiatry. 2014; 4:e459. doi: 10.1038/tp.2014.96

115. Zou F, Chai HS, Younkin CS, Allen M, Crook J, Pankratz VS, Carrasquillo MM, Rowley CN, Nair AA, Middha S, Maharjan S, Nguyen T, Ma L, et al, and Alzheimer's Disease Genetics Consortium. Brain expression genome-wide association study (eGWAS) identifies human disease-associated variants. PLoS Genet. 2012; 8:e1002707. doi: 10.1371/journal.pgen.1002707

116. Webster JA, Gibbs JR, Clarke J, Ray M, Zhang W, Holmans P, Rohrer K, Zhao A, Marlowe L, Kaleem M, McCorquodale DS 3rd, Cuello C, Leung D, et al, and NACC-Neuropathology Group. Genetic control of human brain transcript expression in Alzheimer disease. Am J Hum Genet. 2009; 84:445–58. doi: 10.1016/j.ajhg.2009.03.011

117. Ramasamy A, Trabzuni D, Guelfi S, Varghese V, Smith C, Walker R, De T, Coin L, de Silva R, Cookson MR, Singleton AB, Hardy J, Ryten M, et al, and North American Brain Expression Consortium. Genetic variability in the regulation of gene expression in ten regions of the human brain. Nat Neurosci. 2014; 17:1418–28. doi: 10.1038/nn.3801

118. Zhang B, Gaiteri C, Bodea LG, Wang Z, McElwee J, Podtelezhnikov AA, Zhang C, Xie T, Tran L, Dobrin R, Fluder E, Clurman B, Melquist S, et al. Integrated systems approach identifies genetic nodes and networks in late-onset Alzheimer's disease. Cell. 2013; 153:707–20. doi: 10.1016/j.cell.2013.03.030

119. Kim S, Cho H, Lee D, Webster MJ. Association between SNPs and gene expression in multiple regions of the human brain. Transl Psychiatry. 2012; 2:e113. doi: 10.1038/tp.2012.42

120. Gamazon ER, Badner JA, Cheng L, Zhang C, Zhang D, Cox NJ, Gershon ES, Kelsoe JR, Greenwood TA, Nievergelt CM, Chen C, McKinney R, Shilling PD, et al. Enrichment of cis-regulatory gene expression SNPs and methylation quantitative trait loci among

bipolar disorder susceptibility variants. Mol Psychiatry. 2013; 18:340–46. doi: 10.1038/mp.2011.174

121. Gibbs JR, van der Brug MP, Hernandez DG, Traynor BJ, Nalls MA, Lai SL, Arepalli S, Dillman A, Rafferty IP, Troncoso J, Johnson R, Zielke HR, Ferrucci L, et al. Abundant quantitative trait loci exist for DNA methylation and gene expression in human brain. PLoS Genet. 2010; 6:e1000952. doi: 10.1371/journal.pgen.1000952

122. Shpak M, Hall AW, Goldberg MM, Derryberry DZ, Ni Y, Iyer VR, Cowperthwaite MC. An eQTL analysis of the human glioblastoma multiforme genome. Genomics. 2014; 103:252–63. doi: 10.1016/j.ygeno.2014.02.005

123. Colantuoni C, Lipska BK, Ye T, Hyde TM, Tao R, Leek JT, Colantuoni EA, Elkahloun AG, Herman MM, Weinberger DR, Kleinman JE. Temporal dynamics and genetic control of transcription in the human prefrontal cortex. Nature. 2011; 478:519–23. doi: 10.1038/nature10524

124. Liu C, Cheng L, Badner JA, Zhang D, Craig DW, Redman M, Gershon ES. Whole-genome association mapping of gene expression in the human prefrontal cortex. Mol Psychiatry. 2010; 15:779–84. doi: 10.1038/mp.2009.128
